# Uncovering the significance of expanded CD8^+^ large granular lymphocytes in inclusion body myositis: Insights into T cell phenotype and functional alterations, and disease severity

**DOI:** 10.3389/fimmu.2023.1153789

**Published:** 2023-03-30

**Authors:** Emily McLeish, Anuradha Sooda, Nataliya Slater, Barbara Kachigunda, Kelly Beer, Shereen Paramalingam, Phillipa J. Lamont, Abha Chopra, Frank Louis Mastaglia, Merrilee Needham, Jerome David Coudert

**Affiliations:** ^1^ Centre for Molecular Medicine and Innovative Therapeutics, Murdoch University, Murdoch, WA, Australia; ^2^ Harry Butler Institute, Centre for Biosecurity and One Health, Murdoch University, Murdoch, WA, Australia; ^3^ Perron Institute for Neurological and Translational Science, Nedlands, WA, Australia; ^4^ Department of Rheumatology, Fiona Stanley Hospital, Murdoch, WA, Australia; ^5^ Neurogenetic Unit, Department of Neurology, Royal Perth Hospital, Perth, WA, Australia; ^6^ Institute for Immunology and Infectious Diseases, Murdoch University, Murdoch, WA, Australia; ^7^ School of Medicine, University of Notre Dame, Fremantle, WA, Australia; ^8^ Department of Neurology, Fiona Stanley Hospital, Murdoch, WA, Australia

**Keywords:** inclusion body myositis, T cell large granular lymphocytes, inhibitory natural killer receptors, KLRG1, senescence

## Abstract

**Introduction:**

Inclusion body myositis (IBM) is a progressive inflammatory myopathy characterised by skeletal muscle infiltration and myofibre invasion by CD8^+^ T lymphocytes. In some cases, IBM has been reported to be associated with a systemic lymphoproliferative disorder of CD8^+^ T cells exhibiting a highly differentiated effector phenotype known as T cell Large Granular Lymphocytic Leukemia (T-LGLL).

**Methods:**

We investigated the incidence of a CD8^+^ T-LGL lymphoproliferative disorder in 85 IBM patients and an aged-matched group of 56 Healthy Controls (HC). Further, we analysed the phenotypical characteristics of the expanded T-LGLs and investigated whether their occurrence was associated with any particular HLA alleles or clinical characteristics.

**Results:**

Blood cell analysis by flow cytometry revealed expansion of T-LGLs in 34 of the 85 (40%) IBM patients. The T cell immunophenotype of T-LGL_HIGH_ patients was characterised by increased expression of surface molecules including CD57 and KLRG1, and to a lesser extent of CD94 and CD56 predominantly in CD8^+^ T cells, although we also observed modest changes in CD4^+^ T cells and γδ T cells. Analysis of Ki67 in CD57^+^ KLRG1^+^ T cells revealed that only a small proportion of these cells was proliferating. Comparative analysis of CD8^+^ and CD4^+^ T cells isolated from matched blood and muscle samples donated by three patients indicated a consistent pattern of more pronounced alterations in muscles, although not significant due to small sample size. In the T-LGL_HIGH_ patient group, we found increased frequencies of perforin-producing CD8^+^ and CD4^+^ T cells that were moderately correlated to combined CD57 and KLRG1 expression. Investigation of the HLA haplotypes of 75 IBM patients identified that carriage of the HLA-C*14:02:01 allele was significantly higher in T-LGL_HIGH_ compared to T-LGL_LOW_ individuals. Expansion of T-LGL was not significantly associated with seropositivity patient status for anti-cytosolic 5'-nucleotidase 1A autoantibodies. Clinically, the age at disease onset and disease duration were similar in the T-LGL_HIGH_ and T-LGL_LOW_ patient groups. However, metadata analysis of functional alterations indicated that patients with expanded T-LGL more frequently relied on mobility aids than T-LGL_LOW_ patients indicating greater disease severity.

**Conclusion:**

Altogether, these results suggest that T-LGL expansion occurring in IBM patients is correlated with exacerbated immune dysregulation and increased disease burden.

## 1 Introduction

Sporadic inclusion body myositis (IBM) is a progressive inflammatory disease of skeletal muscles characterized by asymmetric muscle weakness and atrophy that primarily affects the quadriceps, tibialis anterior, finger and forearm flexors (1). The etiology of IBM remains unknown, however consistent findings have documented a significant autoimmune component, with the presence of activated CD8^+^ T cells and Th1-polarized CD4^+^ T cells, elevated pro-inflammatory cytokines, reduced regulatory T cells (2). One of the most prominent histological features of IBM muscles includes endomysial infiltration and invasion of myofibers by CD8^+^ T lymphocytes that exhibit an activated and highly-differentiated phenotype, defined by the re-expression of CD45RA, the loss of secondary lymphoid tissue homing receptor CCR7 and of co-stimulatory receptors CD27 and CD28 (3). Moreover, these cells demonstrate increased effector capabilities as evidenced by increased content in pro-inflammatory cytokine IFN-γ and cytotoxic proteins such as granzymes and perforin (3). The presence of anti-cytosolic-5′-nucleotidase 1A (cN1A) autoantibodies has been reported in 33 to 72% of IBM patients in previous studies (4–8); it has a high sensitivity for IBM because it is only rarely detected in other types of idiopathic inflammatory myopathies (IIM) (9) and also in other autoimmune diseases such as Sjogren’s syndrome and systemic lupus erythematosus (10). Other antibodies reacting to endogenous molecules have been documented in IIM but they lack specificity for IBM, therefore anti-cN1A has a recognized prognostic value in IBM when combined with clinical examination. Additionally, IBM is associated with the presence of certain Human Leukocyte Antigen (HLA) alleles: HLA-DRB1*03:01 and the 8.1 MHC ancestral haplotype (11). Despite these indications of an pathological autoimmune component, conventional immunosuppressive therapies have failed in IBM and no effective treatment options are currently available (12).

In a previous study, a portion of IBM patients was found to be associated with a clonal disorder of CD8^+^ T cells known as T cell large granular lymphocytic leukemia (T-LGLL) (13, 14). T-LGLL is a rare, chronic lymphoproliferative disorder of T cell large granular lymphocytes (T-LGLs); a morphologically distinct sub-population of T cells displaying a large size (15-18 μm), a round or reniform nucleus and an abundance of intracellular cytotoxic granules (15). This type of leukemia is in most instances indolent in nature, predominantly affects elderly people (median age 60 years) (16) and is often accompanied by cytopenias (most frequently neutropenia) and recurrent infections (14). A key molecular hallmark of expanded CD8^+^ T cells in T-LGLL is gain of function mutations leading to constitutive activation of the signal transducer and activator of transcription 3 (*STAT3*) and resulting in increased survival and defective activation-induced cell death (17). Moreover, the association between autoimmunity and T-LGLL is well-documented with approximately 15-40% of cases having concomitant rheumatic disorders including rheumatoid arthritis (RA) with Felty’s syndrome, systemic lupus erythematosus (SLE), Sjogren’s syndrome, and systemic sclerosis (14, 16, 18). Despite these associations, the pathological mechanisms connecting these disorders remain undetermined, although the general consensus is that chronic autoantigen stimulation drives T-LGL differentiation, transformation and persistence into a leukemic state (19).

Initially, a diagnosis of T-LGLL was based on the accumulation of T-LGLs greater than 2x10^9^/L of blood (20). More recently, the utility of this threshold has been debated, with several studies defining T-LGLL using lower cell counts (>0.5x10^9^/L of blood), when appropriate clinical criteria were met (16–18, 21). Furthermore, the classification of T-LGLL as a neoplastic disease is often debated, and despite its reported monoclonal nature, T-LGLL is rarely aggressive like other lymphoproliferative diseases (22).

Alternatively, polyclonal and oligoclonal T-LGL proliferations have been described in numerous other immune-mediated conditions including chronic viral infections, graft-versus-host disease or secondary to cancers (23–25). In these circumstances, T-LGL expansions represent transient “reactive” expansions, and generally resolve within period ranging from a few weeks to months (26). The distinction between reactive T-LGL expansions and T-LGL leukemia based on clonality is still debated as clonal T cell expansions can be observed in patients without neoplastic conditions (27). We, therefore, favored the terminology “expanded T-LGLs or T-LGL_HIGH_” throughout this study.

The phenotype of T-LGL cells resembles normal terminally differentiated CD8^+^ T cells, characterized by the re-expression of CD45RA and the loss of co-receptors CD27 and CD28 (28, 29). Additionally, the great majority of T-LGLs display increased expression of CD57 and CD2, along with the complete or partial loss of CD5 and variable expression of CD7, CD11b, CD11c, CD16, CD94 and CD56(14, 20, 30, 31). Furthermore, in IBM patients, CD8^+^ T cells display increased expression of the killer-cell lectin-like receptor-1 (KLRG1), an inhibitory receptor that, upon engagement by its ligands E and N cadherins, negatively impacts cell proliferation (3). Previous studies have reported the altered expression of CD57 and KLRG1 on CD4^+^ and γδ T cells in chronic inflammatory conditions such as cancer, autoimmune diseases and viral infections (32–36), however, it remains to be determined whether the phenotype changes observed in CD8^+^ T-LGL extend to other T cell subsets and contribute to the overall immune dysregulation in IBM.

This study endeavored to expand our understanding of the phenotypical characteristics of T-LGL in an Australian cohort of IBM patients, and to investigate the clinical implications and possible genetic associations with particular HLA alleles.

## 2 Materials and methods

### 2.1 Sample collection

Peripheral blood and muscle biopsy samples were donated for this study. 85 IBM patients and 56 healthy controls (HC) donated blood; 3 patients donated muscle. Samples were collected between 2018 and 2021. Patients were recruited through the myositis clinics at Murdoch University and the Perron Institute in Western Australia. Diagnoses were established by the treating neurologist, based on clinical and serological findings and in most cases confirmed by a muscle biopsy, fulfilling the European Neuromuscular Centre 2011 criteria for clinicopathological or clinically defined IBM (37). Exclusion criteria for the healthy controls included existence of autoimmune or inflammatory disorders and/or history of immune-modulating medications. Samples were not collected when IBM patients or HC had received a COVID-19 or influenza vaccine within the previous 12 weeks. The Murdoch University Human Research Ethics Committee has approved this study (#2015/111) and all patients were informed and gave written consent to provide samples for research purposes.

### 2.2 Classification of T-LGL expansions in IBM

**Figure 1:**
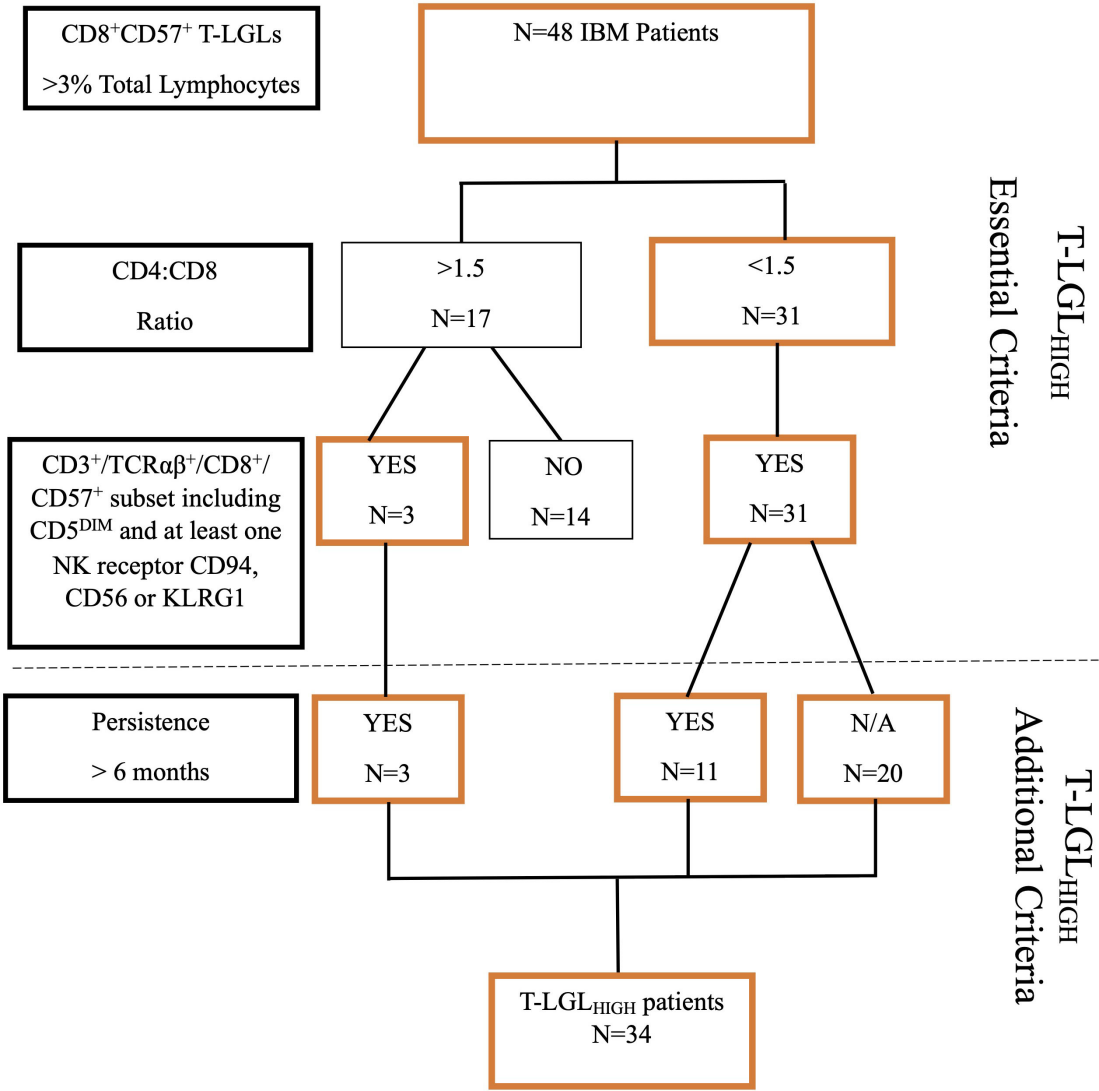
Flowchart of selection criteria for T-LGL_HIGH_ patients.

IBM patients were classified as having an expanded T-LGL population based on the following criteria: 1. number of CD8^+^CD57^+^ T cells constituting greater than 3% of lymphocytes. This value represents two standard deviations (95^th^ percentile) from the mean of our healthy control cohort. 2. An altered CD4:CD8 ratio <1.5, and 3. An aberrant immunophenotype showing increased frequencies of CD3^+^/TCRαβ^+^/CD8^+^ CD57^+^ cells with co-expression including CD5 downregulation and at least one other NK cell-associated receptor (including either CD94, CD56 or KLRG1). The increased frequencies of these surface molecules were again determined as 2 standard deviations above the mean value observed for HC. 4. The proportion of circulating T-LGLs and aberrant phenotype changes that persisted over a 6 month-period. Patients who met at least 3 of these criteria were classified as T-LGL_HIGH_ (**Figure 1**).

### 2.3 Whole blood Wright-Giemsa staining

Peripheral blood smears were prepared using 1 µl of whole blood on epredia™ frosted microscope slide (Thermo Fisher Scientific, Victoria, Australia). The smear was air-dried at room temperature and then fixed in methanol for 5 minutes. Then, the smear was stained with Wright-Giemsa stain (Abcam, Cambridge, United Kingdom) according to the manufacturer’s instructions. Briefly, the smear was first immersed in Wright-Giemsa stain for 5 minutes, rinsed with distilled water and then with phosphate buffer pH 6.8 solution. The stained smear was then air-dried and examined under a microscope at 1000x magnification.

### 2.4 Cell immunophenotyping

Peripheral blood was drawn from the antecubital veins into heparin-lithium coated tubes (Becton Dickinson Bioscience, VIC, Australia). For each flow cytometry staining, 100μl of fresh whole blood were incubated with a panel of fluorochrome-conjugated antibodies: anti-CD3-BV510 (clone UCHT1, BD Bioscience, NJ, USA), anti-CD4-FITC (clone OKT4, BioLegend, CA, USA), anti-CD5-APC-R700 (clone UCHT2, BD Bioscience), anti-CD8-APC-H7 (clone SK1, BD Bioscience), anti-CD56-PE-CF594 (clone B159, BD Bioscience), anti-CD57-BV421 (clone NK-1, BD Bioscience), anti-CD94-PE (clone HP-3D9, BD Bioscience), anti-TCRαβ-PE-Cy7 (clone IP26, BioLegend), anti-KLRG1-APC (clone 13F12F2, Invitrogen, CA, USA). After 30 min incubation with the antibody cocktail, lysing solution (BD Bioscience) was added for red blood cell lysis according to the manufacturer’s recommendations. Samples were washed twice in Phosphate buffer saline (PBS) and resuspended in PBS 2% fetal calf serum (Fisher Biotech, Wembley, WA, Australia) containing count beads (Beckman Coulter, NSW, Australia) for cell count normalization. For proliferation analysis, intranuclear staining for Ki67 was performed: cells were stained for surface markers before fixation using the Transcription Factor Fix/Perm Buffer and transcription factor diluent buffer (BD Bioscience) for 50 min at 4°C, then washed twice with Transcription factor Permeabilization/Wash Buffer (BD Bioscience). The cells were then incubated with anti-Ki67-PerCP 5.5 (clone B56, BD Bioscience) for 50 min, washed three times with Transcription permeabilization buffer (BD Bioscience) and once with PBS. Surface and intracellularly stained cells were resuspended in PBS 2% FCS.

For intracellular staining, cells were incubated for 4 hr at 37°C in a 5% CO_2_ incubator in DMEM 10% FCS with PMA (100 ng/mL), ionomycin (1 µg/mL) and monensin (2 µg/mL) (Sigma Aldrich MA, USA), followed by incubation with a panel of antibodies: anti-CD3-BV510 (clone UCHT1, BD Bioscience), anti-CD4-AF700 (clone OKT4, BioLegend), anti-CD8-APC-H7 (clone SK1, BD Bioscience),anti-CD45RA APC (clone HI100, BioLegend), CD56-PE-CF594 (clone B159, BD Bioscience), for 30 min. Cells were then fixed in BD Cytofix/Cytoperm Fixation/permeabilization solution (BD Bioscience), washed twice with 100μL BD Perm/Wash™ (BD Bioscience), and incubated with anti-IFN-γ PE (clone B27, BioLegend) and anti-Perforin PE-Cy7 (clone B-D48, BioLegend) antibodies for 40min. Cells were then washed three times with Perm/wash solution (BD Bioscience), once with PBS, then resuspended in PBS 2% FCS for flow cytometry acquisition using a Gallios flow cytometer (Beckman Coulter) with at least 100,000 total events captured per sample. Flow cytometry analysis was performed using Flowjo™ 10.5.3 for Windows (Flowjo™ software Inc. USA) and Kaluza™ Analysis 2.1 software (Beckman Coulter, Sydney, NSW, Australia).

The gating strategies used are illustrated as **Supplementary Figures 1**-**4**: 1 (differentiation markers on αβ (A) and γδ (B) T cells), 2 (Ki67 expression in T cells), 3 (phenotype of muscle-isolated T cells) and 4 (intracellular expression of IFN-γ and perforin in T cells).

### 2.5 Leukocyte isolation from muscle

Isolation of leukocytes from muscle biopsies has been described previously by us (38). Briefly, muscle was cut into small pieces and digested using Collagenase P (Sigma-Aldrich) and DNAse I (Sigma-Aldrich). Muscle homogenate was aspirated through a sterile nylon wool-packed 10mL syringe. Filtered cells were washed twice with PBS 2% FCS and separated using a Ficoll (Bio-strategy, Tullamarine, VIC, Australia) gradient. Leukocytes were washed again with PBS 2% FCS, centrifuged, and counted. Immunophenotyping of muscle-isolated leukocytes was performed by staining approximately 25,000 cells with 50μl of antibody mixture, as described above for blood cells.

### 2.6 HLA sequencing

The HLA class I and II allele haplotypes were assessed by Illumina next-generation sequencing by the Institute for Immunology and Infectious Diseases, Murdoch University, accredited by the American Society for Histocompatibility and Immunogenetics (ASHI) and the National Association of Testing Authorities (NATA). The sequencing protocol using locus-specific PCR amplification of genomic DNA was adapted from (39). Briefly, polymorphic regions of HLA class I (A, B, C, exons 2 and 3) were PCR amplified using sample-specific Molecular Indexed (MID-tagged) primers. Amplicons were quantified, normalized, and pooled in equimolar ratios. Sequencing libraries were created and quantified using the Jetseq qPCR Library Quantification Kit (Meridian Bioscience Inc., OH, USA). Samples were sequenced on an Illumina MiSeq platform using the MiSeq V3 600-cycle kit (2×300 base pair reads) (Illumina Inc., CA, USA). Reads were quality-filtered, separated by MID tags and passed through an in-house accredited HLA allele caller software. Alleles were called using the latest IMGT HLA allele nomenclature (40). Allele identity assignation is based on G groups that combine sub-alleles with identical nucleotide sequences across the sequenced exons (41).

### 2.7 Uniform manifold approximation and projection (UMAP) analysis

Flow cytometry raw data were analyzed using Flowjo™ 10.5.3 for Windows (Flowjo™ software Inc. USA). For surface stain analysis, a subset of 50,000 T cells were selected from each donor group, using the *DownSample* Flowjo™ plug-in version 3.3.1, and concatenated into a single matrix prior to (UMAP) analysis (Flowjo™ *UMAP* plug-in version 3.1). A map of markers (TCRαβ, CD4, CD8, CD57, CD5, CD94, CD56 and KLRG1) was generated and clustered according to similar cells’ proximity to one another. For the intracellular analysis of Ki67, a subset of 20,000 T cells were selected from each donor group and concatenated into a single matrix prior to (UMAP) analysis. A map of markers (CD4, CD8, CD57, KLRG1, and Ki67) was generated and clustered according to similar cells’ proximity to one another.

### 2.8 Anti-cN1A measure by ELISA

Anti-cN1A antibodies were measured using a semi-quantitative ELISA adapted from Bundell et al. (42). 96-well plates (Maxisorp, Nunc, Roskilde, Denmark) were coated with 10 µg/ml of cN1A protein (GenScript, NJ, SA) diluted in 50 mM carbonate-bicarbonate buffer (pH 9.6) for 2 hours at ambient temperature. Wells were washed with PBS/0.1% Tween (PBST) and saturated with blocking buffer (PBST/5% skim milk powder) overnight at 4°C. After washing with PBST, 100 µL of patient serum diluted to 1:1,000 in blocking buffer were added in duplicate and incubated for 2 hours at ambient temperature. Then, wells were washed with PBST before adding horseradish peroxidase (HRP)-conjugated anti-human secondary antibodies directed against pan IgG/M/A or IgG or IgM or IgA (Invitrogen, Rockford, IL, USA) and incubating for 1 hour at ambient temperature. Wells were washed as above before revelation by incubation in 50 µL TMB solution (ThermoFisher Scientific, Waltham, MA, USA) for 10 minutes before stopping the reaction with 50 µL of 2M H_2_SO_4_ solution. The absorbance at 450nm was read using a microplate reader FLUOstar Omega (BMG Labtech, Mornington, VIC, Australia). Each plate included a positive control of anti-NT5C1A antibodies purified from the serum of a seropositive patient. Pooled serum from healthy controls diluted 1:1,000 in blocking buffer was used as a negative control. Blank duplicates were obtained by performing all the steps in the absence of a sample. All absorbance values were adjusted by the average blank value. Absorbance obtained for patients’ samples was recorded as a fold change relative to the healthy serum pool. Reference cut-off values correspond to the 99^th^ percentile of the healthy samples (Busselton Population Health Study participants, n = 190). Patients with increased value for at least one antibody isotype were considered seropositive.

### 2.9 Statistical analysis

Statistical analysis was performed using GraphPad PRISM™ (GraphPad Software Inc. *version 8.0.2*, San Diego, CA, USA) and RStudio™ (version V1.4.1717, Integrated Development for R. RStudio, PBC, Boston, MA, USA) (43). Each data set was assessed for normality using the Shapiro-Wilk normality test. A Mann-Whitney U test was used to assess non-parametric data. Wilcoxon matched-pairs sign rank test was used for the paired non-parametric test. Mann-Whitney analysis of variance (ANOVA) and Friedman Test was used for non-parametric and grouped analysis with *post-hoc* test (Dunn’s or Bonferroni) performed for multiple comparisons. Two-way factorial analysis was performed using Scheirer-Ray-Hare test for non-parametric analysis with Tukey *post-hoc* test for multiple comparisons (44, 45). A Spearman’s test was used for non-parametric correlation analysis. Pearson’s Chi-squared test was used for comparison of categorical datasets. To determine the variance of the dataset, a multivariate principal component analysis (PCA) was performed using RStudio version V1.4.1717; correlation analysis was performed using *ggstatplot* package (46). Analysis of HLA data was performed using RStudio V1.4.1717 and the Genentech/MiDAS package (47). Briefly, given that each patient inherits two alleles at each gene locus, allele frequency was calculated as frequency = n (allele occurrence count)/(2 * number of alleles detected). For final analysis, only alleles measured at a frequency above 2% were included. *P*-values < 0.05 were considered statistically significant. *: P≤0.05, **: P≤0.01, ***: P≤0.001, ****: P≤0.0001, NS: not significant.

## 3 Results

### 3.1 Cohort demographics and hematological information

Cohort demographics and blood lymphocyte counts are summarized in **Table 1**; more detailed demographics and clinical information are reported in **Supplementary Table 1** and **Supplementary Figure 5**. Although both the IBM patients and the HC groups are comprised of older adults, the average age was 5 years more in the patient group than the control group. The proportion of males and females was not statistically different between the patient and control groups (IBM= 47:38 vs HC= 27:29, P=0.39). The CD4^+^:CD8^+^ T cell ratio was lower in the blood of IBM patients than in HC (HC=2.8 vs IBM=2.0, P=0.04. No significant differences were observed for blood counts of CD4^+^ (0.32x10^9^/L in IBM, 0.39x10^9^/L in HC, P=0.078), or CD8^+^ T cells (0.16x10^9^/L in IBM, 0.1x10^9^/L in HC, P=0.17) (**Table 1**).

**Table 1:** Demographic and hematological information in IBM and HC groups.

	HC	IBM	P-Value
**Number**	56	85	
**Age years Median** **(range)**	68 (47-87)	73 (41-96)	1.8x10^-3^
**Males: Females** **numbers (%)**	27:29 (48.20/51.8%)	47:38 (55.29/44.71%)	0.39
**Lymphocyte count/L blood median (range)**	0.93x10^9^ (0.2-2.9x10^9^)	1.03x10^9^ (0.2-5.08x10^9^)	0.48
**T cells/L blood median** **(range)**	0.57x10^9^ (0.13-1.48x10^9^)	0.56x10^9^ (0.11-3.57x10^9^)	0.47
**CD4^+^ count/L of blood median (range)**	0.39x10^9^ (0.12-0.97x10^9^)	0.32x10^9^ (0.03-1.69x10^9^)	0.078
**CD8^+^ count/L of blood median (range)**	0.1x10^9^ (0.041-0.68x10^9^)	0.16x10^9^ (0.023-3.12x10^9^)	0.17
**CD4:CD8 ratio median** **(range)**	2.8 (0.3-15.19)	2.0 (0.2-18.01)	0.04

### 3.2 IBM patients demonstrate increased frequency of CD8^+^CD57^+^ T-LGLs

**Figure 2:**
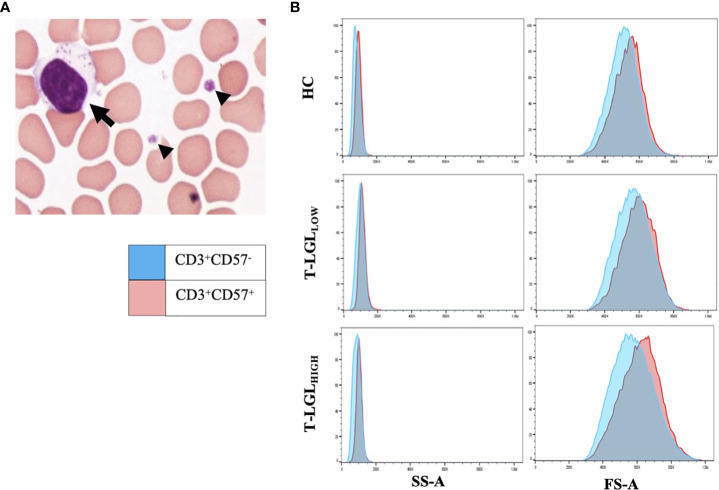
LGLs are enlarged cells with granular content. **(A)** Representative LGL cell showing increased size (15μm) with multiple cytotoxic granules (arrow). Small arrowheads indicate platelets. The image was taken at 100x magnification. **(B)** Forward (FS-A) and Side (SS-A) Scatter area measures of representative blood samples from the HC, T-LGL_LOW_ and _-HIGH_ patient groups. Comparative SS-A and FS-A parameter measures are illustrated for the CD3^+^CD57^+^ (red histograms) and the CD3^+^CD57^-^ cells (blue histograms) subsets.

We performed blood smears and detected in a fraction of IBM patients the presence of blastic lymphocytes containing cytoplasmic granules, which matches the description of T-LGL morphology (**Figure 2A**). Comparative analysis of the forward and side scatter flow cytometry parameters, which measure the cell size and granularity respectively, showed that CD57^+^ cells displayed higher values for each of these parameters than their CD57^-^ counterparts (**Figure 2B**). The normal proportion of T-LGLs reported in adults is estimated between 10 to 15% of total PBMCs (48). However, in our HC cohort, we consistently found less CD8^+^CD57^+^ T-LGLs within total lymphocytes (median 1.10%; range=0-11%). According to our T-LGL inclusion criteria defined in the *methods section*, we identified 34 out of 85 IBM patients (40%) as T-LGL_HIGH_. We found in T-LGL_HIGH_ patients that the median proportion of CD8^+^CD57^+^ T-LGL in total lymphocytes was 9.12%, which is comparatively greater than in T-LGL_LOW_ and HC (1.23% and 1.10%, P≤0.0001 respectively; **Table 2**).

**Table 2:** Number of peripheral blood lymphocytes in HC and IBM T-LGL_LOW_ and T-LGL_HIGH_ donor groups.

	HC	IBM T-LGL_LOW_	IBM T-LGL_HIGH_	Kruskal-Wallis ANOVA
**NUMBER**	56	51	34	
**LYMPHOCYTE COUNT/L BLOOD MEDIAN (RANGE)**	0.98x10^9^ (0.22-2.61x10^9^)	0.88x10^9^ (0.25-3.79x10^9^)	1.24x10^9^ (0.29-5.08x10^9^)	0.069
**CD8^+^CD57^+^ (/L BLOOD) MEDIAN (RANGE)**	0.012x10^9^ (1.16x10^5^-0.13x10^9^)	0.0086x10^9^ (3.26x10^5^-0.21x10^9^)	0.108x10^9^ (0.02-0.70x10^9^)	<0.0001
**% CD8^+^CD57^+^ IN TOTAL LYMPHOCYTES MEDIAN; 95^TH^ PERCENTILE (RANGE)**	1.10; 3 (0-11)	1.23; 2.4 (0.03-6.94)	9.12; 14.8 (3.36-44.27)	<0.0001

Next, we assessed whether these T-LGL expansions persisted over time. We conducted a longitudinal analysis in 42 (14 T-LGL_HIGH_ and 23 T-LGL_LOW_) of the 85 IBM patients. The initial and the latest collection dates were separated by 599 days on average for the T-LGL_HIGH_ group and 544 days for the T-LGL_LOW_ donor group. During that period, the proportions of CD8^+^CD57^+^ cells persisted in all 14 IBM T-LGL_HIGH_ patients (**Supplementary Table 1**). We found 6 T-LGL_LOW_ patients who displayed changes within their CD8^+^ T cell population. Three patients had increased proportions of CD8^+^ T cells among total T cells, but without changes in CD57 expression. Another patient had an increased proportion of CD8^+^CD57^+^ T cells, but no overall increase in total CD8^+^ T cell frequency. Two patients had increased proportion of CD8^+^CD57^+^ T cells with aberrant expression of CD5, CD94, CD56 and KLRG1. However, none of these patients displayed changes that were sufficient to warrant re-classification as T-LGL_HIGH_ during this observation period.

### 3.3 The immunophenotype features of expanded CD8^+^ T-LGLs in IBM

**Figure 3:**
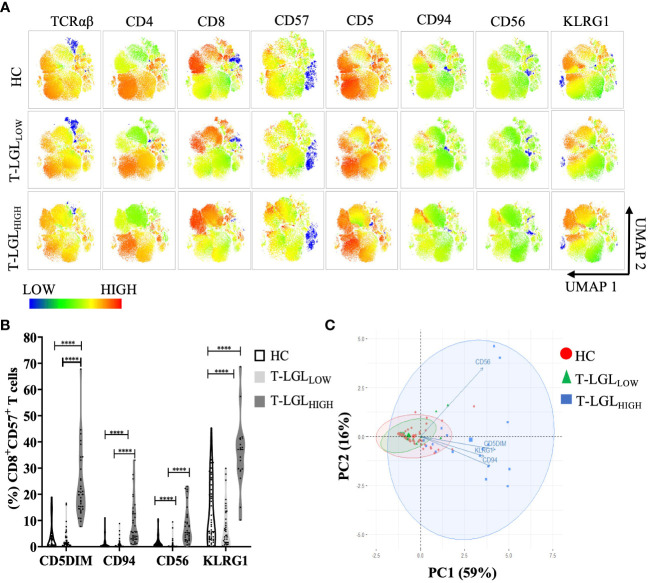
Immunophenotyping of CD3^+^ T cells in HC and IBM patients. **(A)** UMAP heat-map analysis. We selected a total of 50,000 T cells per donor group (HC n=54, T-LGL_NEG_ n=42, T-LGL_POS_ n=17), and concatenated into a single matrix. T cells were identified based on the signal expression intensity of the phenotypical markers TCRαβ, CD4, CD8, CD57, CD5, CD94, and KLRG1. **(B)** Median frequency of surface markers in CD8^+^CD57^+^ T cells. Statistical analysis was performed individually for each surface antigen using the Kruskal-Wallis ANOVA with Dunn’s *post-hoc* test for multiple comparisons. Healthy controls (n=56), IBM T-LGL_LOW_ (n=51), and IBM T-LGL_HIGH_ (n=34). *KLRG1 was analyzed in HC (n=54), IBM T-LGL_LOW_ (n=41), and IBM T-LGL_HIGH_ patients (n=17).* The full comparison of the descriptive statistics for each surface marker is shown in **Supplementary Table 2**. **(C)**. Principal Component Analysis of immune cell phenotypes in CD8^+^CD57^+^ T cells. Each immunophenotype was visualized in 2 dimensions (PC1 and PC2). **** : P < 0.0001.

To study the immunophenotype of CD8^+^ T-LGLs in IBM, we analyzed the expression pattern of surface molecules CD5, CD56, CD94 and KLRG1 on CD8^+^CD57^+^ T cells by flow cytometry. The unsupervised UMAP heat map analysis revealed that in T-LGL_HIGH_ patients, a large cluster of CD8^+^ T cells could be clearly identified, with a moderate to high intensity of CD57 and KLRG1 expression, a moderate expression of CD94 and CD56, and a low to moderate expression of CD5. These expression changes were more pronounced than those observed in the T-LGL_LOW_ patients and the HC (**Figure 3A**). All evaluated surface molecules in the CD8^+^CD57^+^ populations were found to be significantly altered (P≤0.0001) in the T-LGL_HIGH_ patients compared to both the T-LGL_LOW_ patients and HC groups. However, the cell profile was not significantly different for any of these surface molecules between T-LGL_LOW_ patients and HC (**Figure 3B**; **Supplementary Table 2** and **Supplementary Figure 6**).

We performed a correlation matrix analysis for all the markers examined in CD8^+^ T cell subsets for each donor groups. We found the largest extent of marker correlation in the HC and the T-LGL_LOW_ groups whilst most of these correlations were not found in the T-LGL_HIGH_ group (**Supplementary Figure 7**, four bottom rows). This difference may result from the highest variability of marker expression within this donor group which reduced the coefficient of correlation, and from a smaller sample size which negatively impacted the power analysis and P-values. To explore the relationship between these markers in more depth, we investigated the combined expression of CD94, CD56, KLRG1 and CD5 in CD8^+^CD57^+^ T cells. We used a principal component analysis (PCA) to reduce the dimensionality of immunophenotyping data (**Figure 3C**). The first two principal components (PC1 and PC2) explained 75% of the total variance in the data and were used to develop our model. The biplot analysis demonstrated the PC1 positive loadings that consisted of the CD5^DIM^ CD94^+^ KLRG1^+^ phenotypes whereas the PC2 positive loadings comprised expression of CD56. This modelling adequately separated the three groups into HC, T-LGL_LOW_ and T-LGL_HIGH_ IBM patients (**Figure 3C**). The T-LGL_HIGH_ patient group displayed the greatest data variability as indicated by the considerable ellipse size.

Additionally, the magnitude of the PCA correlations between individual marker variations was assessed by analyzing the relative distances between the variables. We found that the CD94^+^, KLRG1^+^ and CD5^DIM^ phenotypes were closely clustered and therefore showed strong correlation, whereas CD56 changes were not correlated with changes of the three other molecules. Accordingly, the immunophenotype of T-LGLs in IBM was typically CD3^+^, TCRαβ, CD8^+^, CD57^+^, CD5^DIM^, KLRG1^+^, CD94^+^ with variable expression of CD56.

### 3.4 IBM-associated T-LGL expansion demonstrates limited proliferative potential

**Figure 4:**
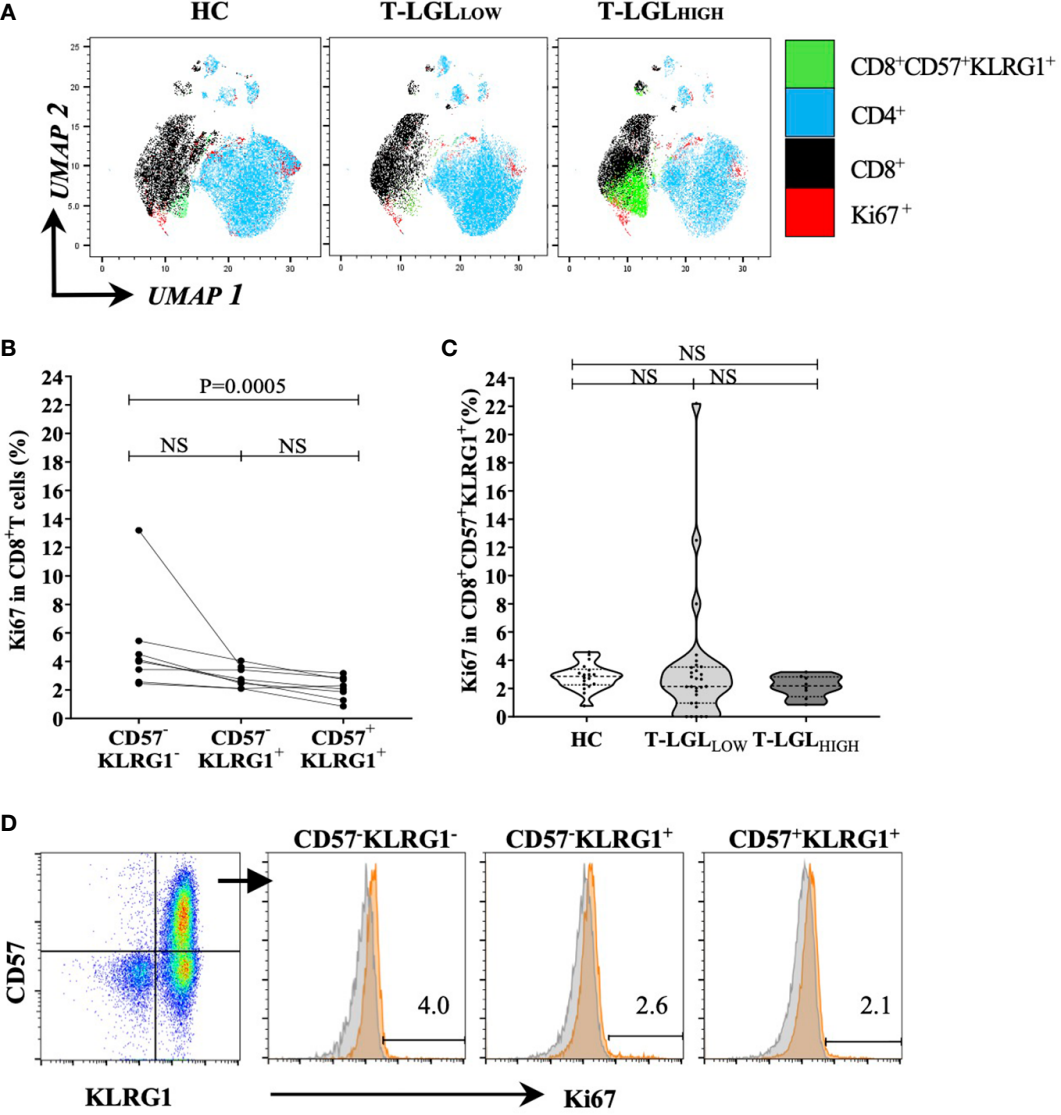
Proportion of Ki67 between HC and IBM. **(A)** UMAP analysis. we selected a total of 20,000 T cells per donor group (HC n=19, T-LGLNEG n=31, T-LGLHIGH n=8), and concatenated into a single matrix. T cells were identified based on the number of cells expressing phenotypical markers CD4, CD8, CD57, KLRG1 and Ki67. **(B)** Percentage of Ki67-expressing CD8+ T cells in the CD57-/+, KLRG1-/+ subsets in IBM T-LGLHIGH patients (n=8). **(C)** Percentage of CD8+ CD57+KLRG1+ T cells expressing Ki67 in the HC and IBM T-LGLLOW and T-LGLHIGH donor groups. Statistical analysis was performed using Friedman test with Dunn’s post-hoc test for multiple comparisons. The P-values are indicated; NS= Not Significant. **(D)** Representative flow cytometry biplot and histograms showing the median frequency of Ki67 in the three CD57-/+, KLRG1-/+ populations.

Considering that both CD57 and KLRG1 were the most predominantly expressed surface molecules on CD8^+^ T-LGLs, we selected this marker coexpression profile to further investigate the biology of this cell population. We analyzed the nuclear protein Ki67 as a marker of proliferation in the CD8^+^CD57^+^KLRG1^+^ population. We performed an unsupervised UMAP analysis to visualize the Ki67-expressing cells within the three donor groups. We detected a small cluster of Ki67^+^ cells within the CD8^+^CD57^+^KLRG1^+^ population in all donor groups whilst more apparent in the T-LGL_HIGH_ group (**Figure 4A**). However, taking into account that the size of the CD8^+^CD57^+^KLRG1^+^ subset varied between groups, the proportion of Ki67^+^ cells within this subset did not significantly differ between the three donor groups (**Figure 4B**).

Next, we investigated in the T-LGL_HIGH_ donor group, the proportion of cells within the CD8^+^CD57^-^KLRG1^-^ (less differentiated), the CD8^+^CD57^-^KLRG1^+^ (intermediate) and the CD8^+^CD57^+^KLRG1^+^ (highly differentiated) subsets that expressed Ki67. We found that this proportion was low in all subsets (most measures within the 1-6% range) and that it was consistently greater in the less differentiated than in the late differentiated cells (median frequency= 4.05% *vs* 2.18%, P=0.0005; **Figures 4C, D**). Similar analysis of the proportion of CD8^+^CD57^+^ T cells expressing Ki67 in the T-LGL_LOW_ and HC groups did not reveal significant differences between the stages of differentiation (**Supplementary Figure 8**).

These results suggest that, as these T cells differentiate into T-LGLs, their proliferative activity decreases, and a small fraction retains active proliferation.

### 3.5 Phenotype alterations in CD4^+^ and γδ T cell subsets in the context of T-LGL disorder

We examined whether the phenotype alterations observed in CD8^+^ T cells also extended to CD4^+^ and γδ T cells in blood. Because of the limited number of fluorochromes that could be simultaneously detected by our flow cytometer, we could not include an anti-TCRγδ antibody in the panel for positive gating and used the CD3^+^TCRαβ^-^ population as a surrogate. In order to validate the suitability of this strategy, we compared the frequency of TCRγδ cells obtained by this approach with the frequency measured using an anti-TCRγδ antibody in the same samples (**Supplementary Figure 1C**). In addition, we applied a correlation analysis to further confirm that we percentages obtained using these two methods were comparable (**Supplementary Figure 1D**).

**Figure 5:**
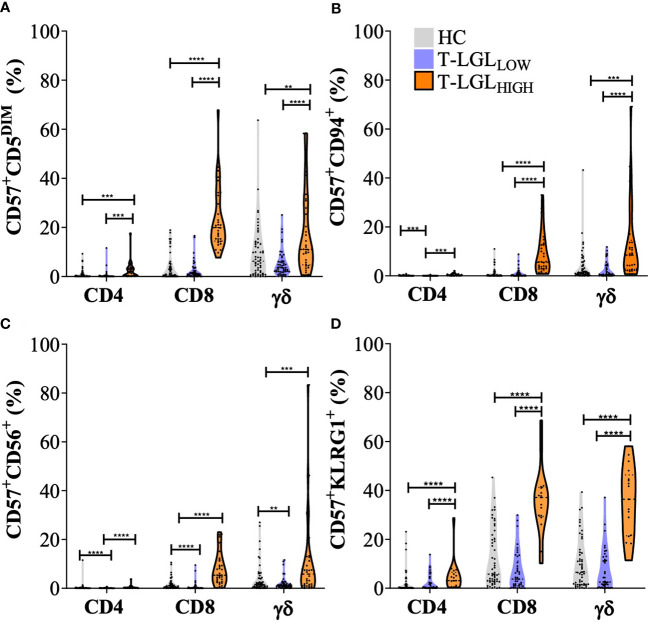
Immunophenotyping of T cell populations in healthy controls, IBM T-LGL_LOW_, and IBM T-LGL_HIGH_. Frequency of CD57^+^CD5^DIM^ cells **(A)**, CD94^+^ T cells **(B)**, CD56^+^ T cells **(C)**, and KLRG1^+^ cells **(D)** in CD4^+^, CD8^+^ and γδ T cells (identified as CD3^+^, TCRαβ^-^ cells). Statistical analysis was performed individually for each surface antigen using the Scheirer–Ray–Hare analysis for Factor analysis with Tukey *post-hoc* test for multiple comparisons. Healthy controls (n=56, grey violin plots), IBM T-LGL_LOW_ (n=51, purple violin plots), and IBM T-LGL_HIGH_ (n=34, orange violin plots). ** : P < 0.01, *** : P < 0.001, **** : P < 0.0001. *KLRG1 was analyzed in HC (n=54), IBM T-LGL_LOW_ (n=41), and IBM T-LGL_HIGH_ patients (n=17).* The full comparison of the descriptive statistics for each surface marker and representative flow cytometry plots are shown in **Supplementary Table 2**.

We applied a two-way analysis of variance (ANOVA) using the Scheirer-Ray Hare method to address differences in CD5, CD94, CD56 and KLRG1 expression within the CD4^+^CD57^+^ CD8^+^CD57^+^ and TCRγδ^+^CD57^+^ T cell subsets and between donor groups (HC, T-LGL_LOW_ and T-LGL_HIGH;_ **Figure 5**). Within the CD4^+^ T cell subset, the frequency of CD57 cells co-expressing CD94, CD56 and with low CD5 expression was greater in T-LGL_HIGH_ than in T-LGL_LOW_ patients and HC. However, these alterations were detected in a considerably smaller fraction of the CD4^+^ T cells than in their CD8^+^ counterparts (**Figure 5A**). Out of all the surface molecules analyzed, KLRG1 was the most prominently expressed in the CD4^+^ subset. Like CD8^+^ T cells, the frequency of CD57^+^KLRG1^+^-expressing CD4^+^ T cells was greater in T-LGL_HIGH_ patients than in HC (median= 3.08% *vs* 0.24%, P=0.0002), and in T-LG_LOW_ patients (median= 3.08% *vs* 0.36%, P=0.0018). Interestingly, similar phenotype changes were observed in γδ T cells, and were more pronounced in T-LGL_HIGH_ than in T-LGL_LOW_ patients and HC.

Finally, the proportion of CD57^+^ T cells with altered expression of CD5, CD94, CD56 and KLRG1 were significantly different in CD8^+^ (TCRαβ^+^) and in TCRγδ^+^ cells in T-LGL_HIGH_ patients compared to other donor groups, while the proportion of CD4^+^ T cells displaying any of these changes was considerably lower.

We performed a correlation matrix analysis for all the markers examined in the CD4^+^, CD8^+^ and γδ T cell populations, and for each donor groups. We found the largest extent of marker expression correlation between γδ T cells and CD8^+^ T cells, but not CD4^+^ T cells, in the HC and the T-LGL_LOW_ groups, while only some of these correlations were observed in the T-LGL_HIGH_ group (**Supplementary Figure 7**).

These results indicated that CD57^+^ T cells exhibited increased expression of CD94, CD56, KLRG1 and decreased CD5 in T-LGL_HIGH_ compared to T-LGL_LOW_ IBM patients and HC. In the T-LGL_HIGH_ group, we found greater proportions of cells that displayed expression changes of multiple differentiation markers within the CD8^+^ and TCRγδ^+^ T cell populations, while increased proportion of CD57^+^KLRG1^+^ cells were detected to a lower extent within the CD4^+^subset.

### 3.6 Comparison of CD8 T cell phenotype in IBM muscle and blood

**Figure 6:**
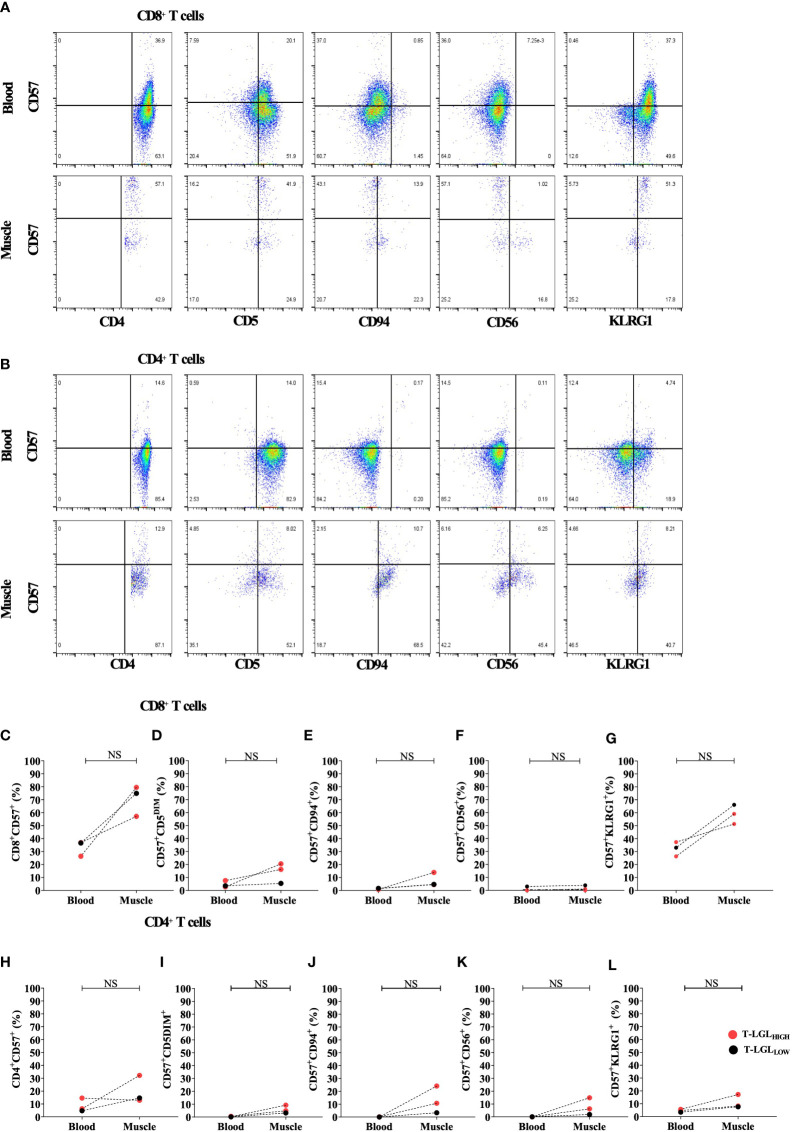
Comparison of CD8^+^ and CD4^+^ T cell phenotype in blood and muscle. Representative Flow cytometry plots gated on **(A)** and CD4^+^ T cells **(B)**. Comparison of the proportion in blood and muscle of CD57^+^ in CD8^+^ **(C)** and CD4^+^ **(H)** T cells, and of CD5^DIM^, CD94^+^, CD56^+^ and KLRG1^+^ in CD8^+^CD57^+^ **(D–G)** and in CD4^+^CD57^+^ **(I–L)** cells. Statistical analysis was performed using one-tailed Wilcoxon matched pairs signed rank test T-LGL_HIGH_ n=2, T-LGL_LOW_ n=1). NS, Not Significant.

We compared the phenotype of CD8^+^ (**Figure 6A**) and CD4^+^ (**Figure 6B**) T cells isolated from muscle and blood samples collected on the same day from two T-LGL_HIGH_ and one T-LGL_LOW_ IBM patients. We found that the frequency of CD8^+^ T cells expressing CD57 was consistently greater in the muscle environment. Indeed, the median frequency of the CD57^+^ fraction represented 74.9% vs 36.6% in muscle vs blood respectively (**Figure 6C**). We found in the two T-LGL_HIGH_ IBM patients that the proportion of CD57^+^ CD5^DIM^ was more elevated in the muscle than in the blood (16.2% vs 7.59% and 20.5% vs 2.8%). Similarly, in the T-LGL_LOW_ patient the proportion of CD57^+^ CD5^DIM^ cells was also higher in muscle than in blood (16.2% vs3.55%). Overall, the proportion of CD57^+^ CD5^DIM^ T cells were not significantly different in muscle and blood (P=0.25; **Figure 6D**).

The proportion of CD57^+^CD94^+^ in CD8^+^ T cells showed an increased trend in muscle compared to blood, however not statistically validated due to the limited number of samples (**Figure 6E**). The fraction of the CD57^+^CD56^+^ subset within CD8^+^ T cells were very similar in muscle and blood with a difference not exceeding 1% across all the patients tested (**Figure 6F**). Furthermore, in all three patients the frequency of CD8^+^ T cells with a CD57^+^KLRG1^+^ phenotype was consistently 2 times greater in muscle than in blood. However, this was not statistically significant (P=0.25; **Figure 6G**). Similar phenotype alterations were also observed on CD4+ T cells (**Figures 6H–L**). Overall, both CD4^+^ and CD8^+^ T cells demonstrated exacerbated alterations of their surface molecule profile in muscle compared to blood with the exception of CD56 that remained inconsistently detected.

### 3.7 Evidence of elevated inflammatory and cytotoxic mediator content in both CD8^+^ and CD4^+^ T cell subsets in T-LGL_HIGH_ IBM patients

**Figure 7:**
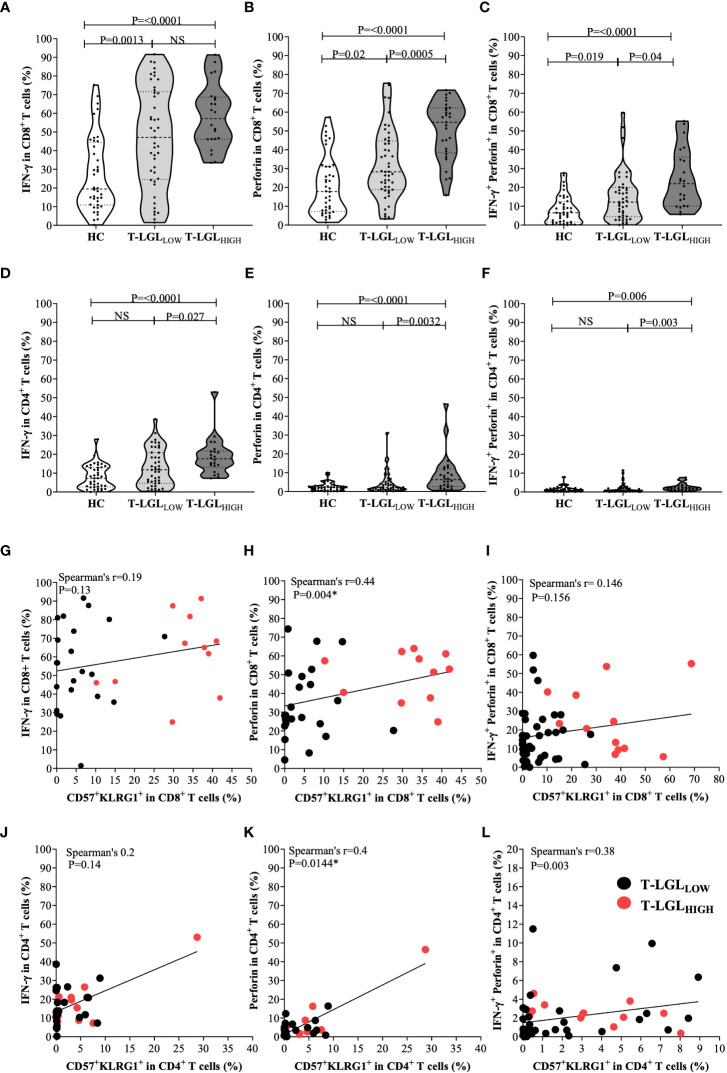
Inflammatory and cytotoxic potential of CD8^+^ and CD4^+^ T cells in healthy controls, IBM T-LGL_LOW_, and IBM T-LGL_HIGH_. **(A)** Median frequency of IFN-γ in CD8^+^ T cells HC (n=40), IBM T-LGL_LOW_ (n=45), and IBM T-LGL_HIGH_ (n=22). **(B)** Median frequency of perforin in CD8^+^ T cells HC (n=40), IBM T-LGL_LOW_ (n=47), and IBM T-LGL_HIGH_ (n=29). **(C)** Median frequency of IFN-γ ^+^perforin^+^ in CD8^+^ T cells HC (n=40), IBM T-LGL_LOW_ (n=45), and IBM T-LGL_HIGH_ (n=22). **(D)** Median frequency of IFN-γ in CD4^+^ T cells HC (n=40), IBM T-LGL_LOW_ (n=45), and IBM T-LGL_HIGH_ (n=22). **(E)** Median frequency of perforin in CD4^+^ T cells HC (n=40), IBM T-LGL_LOW_ (n=47), and IBM T-LGL_HIGH_ (n=29). **(F)** Median frequency of IFN-γ^+^ perforin^+^ in CD4^+^ T cells HC (n=40), IBM T-LGL_LOW_ (n=45), and IBM T-LGL_HIGH_ (n=22). Statistical analysis was performed using Kruskal-Wallis ANOVA and Bonferroni *post hoc* test for multiple comparisons. The P-values are indicated; NS, Not Significant. **(G)** Spearman’s correlation between the proportion of CD8^+^ IFN-γ^+^ and CD57^+^KLRG1^+^ cells in T-LGL_LOW_ (n=23) and T-LGL_HIGH_ patients (n=11). **(H)** Spearman’s correlation between the proportion of CD8^+^ perforin^+^ and CD57^+^KLRG1^+^ cells in T-LGL_LOW_ (n=23) and T-LGL_HIGH_ patients (n=11). **(I)** Spearman’s correlation between the proportion of CD8^+^IFN-γ^+^ perforin^+^ and CD57^+^KLRG1^+^ cells in T-LGL_LOW_ (n=38) and T-LGL_HIGH_ patients (n=12). **(J)** Spearman’s correlation between the proportion of CD4^+^ IFN-γ^+^ and CD57^+^KLRG1^+^ cells in T-LGL_LOW_ (n=23) and T-LGL_HIGH_ patients (n=11). **(K)** Spearman’s correlation between the proportion of CD4^+^perforin^+^ and CD57^+^KLRG1^+^ cells in T-LGL_LOW_ (n=23) and T-LGL_HIGH_ patients (n=11). **(L)** Spearman’s correlation between the proportion of CD4^+^ IFN-γ^+^ perforin^+^ and CD57^+^KLRG1^+^ cells in T-LGL_LOW_ (n=38) and T-LGL_HIGH_ patients (n=12).

To further elucidate the pathogenic role of T-LGLs in IBM, we analyzed the IFN-γ and perforin content in CD8^+^ and CD4^+^ T cell populations (**Figures 7A–F** respectively). In CD8^+^ T cells, we found higher proportions of IFN-γ producing cells in both the T-LGL_HIGH_ and the T-LGL_LOW_ IBM patient groups relative to HC (median=57.1% and 47.1% *vs* 19.5% respectively, P<0.0001 and P= <0.0013; **Figure 7A**). However, no difference was observed between the IBM T-LGL_HIGH_ and T-LGL_LOW_ patients (P=0.23). The proportion of CD8^+^ T cells expressing perforin was more elevated in the T-LGL_HIGH_ individuals compared to HC (median= 54.6% *vs* 17.8%, P<0.001) as well as compared to T-LGL_LOW_ patients (median= 54.6 *vs* 28.3%, P=0.0005; **Figure 7B**). The difference in proportion of IFN-γ and perforin-coexpressing CD8^+^ T cells between groups closely resembled the results observed with perforin alone (**Figure 7C**).

We used an alternate gating strategy to discriminate within CD3^+^ T cells the CD45RA-negative (effector memory and central memory cells) and -positive (naïve and TEMRA cells) fractions, in which we assessed the IFN-γ and perforin content. This strategy provided more precise insights into the CD8^+^CD57^+^ T-LGL subset which is contained within the TEMRA subset. As shown in **Supplementary Figure 9**, the frequency of IFN-γ producing CD8^+^ T cells was not statistically different in the CD45RA-negative and positive fractions in both T-LGL patient groups. Conversely in the HC group, IFN-γ production was higher in the CD45RA-negative fraction, denoting a higher inflammatory potential by memory CD8 T cells than by the naïve/TEMRA fraction. With regard to perforin production, the frequency of CD8^+^ T cells armed for cytotoxicity was significantly higher within the CD45RA^+^ fraction in all three donor groups, with the most significant P-value out of the three donor groups measured in the T-LGL_LOW_ while a lower significance was found in the T-LGL_HIGH_. This possibly resulted from a much smaller analytical power due to the smaller number of samples rather than from a smaller change in content. Therefore, the increased percentage of perforin-producing CD8^+^ T cells in the LGL_HIGH_ group (**Figure 7B**) was likely to result from a combination of a higher representation of the CD57^+^ subset within the CD8^+^ T cells and of from an increased proportion of perforin-producing cells.

In CD4^+^ T cells, we noted that T-LGL_HIGH_ patients possessed comparatively higher proportions of IFN-γ-producing cells than HC (median 17.6% *vs* 7.3%, P≤0.0001), and T-LGL_LOW_ patients (median= 17.6% *vs* 11.8%, P0.027; **Figure 7D**). Moreover, more CD4^+^ T cells produced perforin in T-LGL_HIGH_ than in T-LGL_LOW_ patients (median= 6.9% *vs* 2.1%, P=0.0032) (**Figure 7E**). A higher proportion of perforin-expressing CD4^+^ T cells was measured in T-LGL_HIGH_ compared to HC (median= 6.88% *vs* HC 2.215%, P≤0.0001). Again, we found that the proportions of CD4^+^ T cells coexpressing IFN-γ and perforin in the three donor groups mimicked those of the cells expressing perforin alone (**Figure 7F**).

We applied the same gating strategy based on CD45RA to CD4^+^ T cells as we did for CD8^+^ T cells. We found consistently in all three donor groups that the IFN-γ producing cells were significantly more frequent within the CD45RA^-^ than in the CD45RA^+^ cells, whereas the frequency of perforin-positive cells was higher in the CD45RA^+^ fraction. Although statistically significant, only modest changes for perforin were measured in CD4^+^ T cells from HC and LGL_LOW_ patients while they were more evident in the LGL_HIGH_ group.

Lastly considering that antibody panels for the intracellular content and surface phenotyping were distinct, we were not able to directly demonstrate a direct link between the changes. To overcome this limitation, we retrospectively compared in each sample the proportion of IFN-γ and perforin-producing CD8^+^ and CD4^+^ T cells with the proportion of the CD57^+^KLRG1^+^ subset that has previously been reported to correspond to the most differentiated T cells in IBM (3). We found that the proportion of CD57^+^KLRG1^+^ did not significantly correlate to the proportion of CD8^+^ IFN-γ^+^ (Spearman’s r=0.19, P=0.13; **Figure 7G**). The proportion of CD57^+^KLRG1^+^ and CD8^+^perforin^+^ cells did reveal a modest correlation (Spearman’s r=0.46, P=0.0028; **Figure 7H**). However, no correlation was found between this subset and the proportion of IFN-γ and perforin coexpressing CD8^+^ T cells (Spearman’s r=0.146, P=0.156; **Figure 7I**). Similar results were also observed within the CD4^+^ T cells where the proportion of IFN-γ^+^ did not correlate with CD57^+^KLRG1^+^ (Spearman’s r=0.2, P=0.08) while a modest correlation between the proportion of both perforin^+^ and IFN-γ^+^perforin^+^ CD4^+^ T cells with CD57^+^KLRG1^+^ was found (Spearman’s r=0.4, P=0.0144 and Spearman’s r=0.38, P=0.003, respectively; **Figures 7J–L**).

In summary, T-LGL_HIGH_ patients significantly differ from both T-LGL_LOW_ and HC by higher proportion of CD8^+^ T cells with elevated perforin content, and of CD4^+^ T cells producing both perforin and IFN-γ. Both CD8^+^ and CD4^+^ CD57^+^KLRG1+ T cells were likely producers of perforin.

### 3.8 Investigating HLA association with T-LGL in IBM

**Figure 8:**
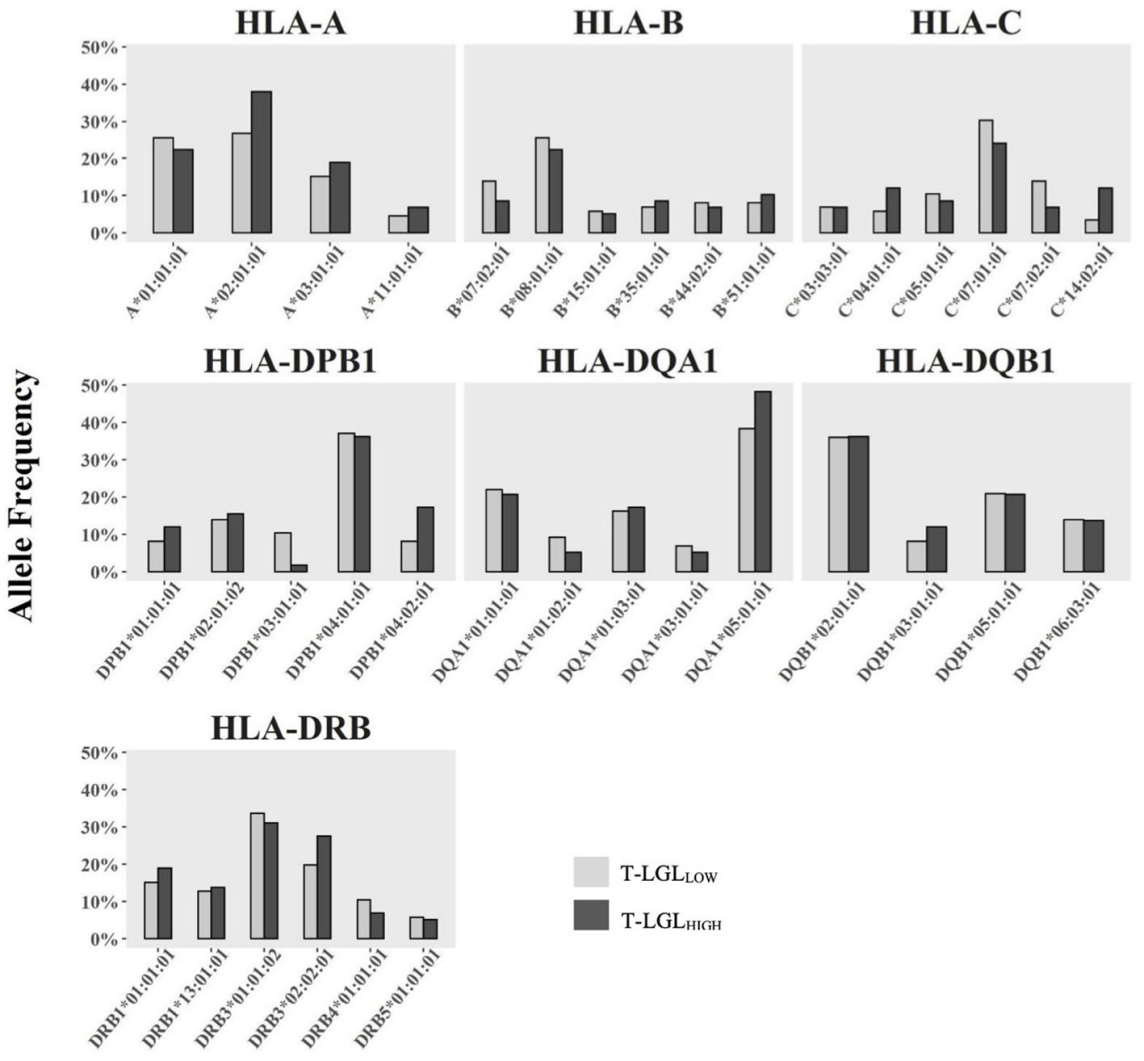
Comparison of HLA Class I and Class II allele frequency (%) between IBM T-LGL_LOW_ and IBM T-LGL_HIGH_ patients. Comparative analysis of HLA allele frequencies in 43 T-LGL_LOW_ and 29 T-LGL_HIGH_IBM patients was performed using the Fisher’s exact test. Minor alleles present at a frequency less than 5% in either group were omitted.

We investigated whether particular HLA alleles may be associated with the occurrence of CD8^+^ T-LGL expansion in IBM. We determined the HLA haplotypes of 72 IBM patients (29 T-LGL_HIGH_ and 43 T-LGL_LOW_ **Table 3** and **Figure 8**). The most relevant alleles, present at a frequency >5% in either of the two patient groups are presented in **Table 3** while the full list of allele frequencies is provided in **Supplementary Table 3**. Analysis of allele frequency identified one allele, HLA-C*14:02:01, which was over-represented in the T-LGL_HIGH_ compared to the T-LGL_LOW_ patients (12.07% *vs* 3.49%, OR=4.24, P =0.05) suggesting of a possible HLA-related genetic role of T-LGL expansion in IBM.

**Table 3:** HLA class I allele frequency in T-LGL_HIGH_ and T-LGL_LOW_ IBM patients.

	HLA Allele	% In total IBM N=144	% In T-LGL_HIGH_ (N=29)	% In T-LGL_LOW_ (N=43)	OR (95^th^ percentile)	p.value
**1**	C*14:02:01G	6.94%	12.07%	3.49%	4.24 (21.24)	0.05
**2**	DPB1*04:02:01G	11.81%	17.24%	8.14%	2.71 (8.57)	0.08
**3**	DPB1*03:01:01G	6.94%	1.72%	10.47%	0.17 (0.90)	0.10
**4**	DQA1*05:01:01G	42.36%	48.28%	38.37%	2.17 (6.24)	0.12
**5**	A*02:01:01G	31.25%	37.93%	26.74%	1.69 (3.59)	0.16
**6**	C*04:01:01G	8.33%	12.07%	5.81%	2.42 (9.06)	0.17
**7**	C*07:02:01G	11.11%	6.90%	13.95%	0.50 (1.42)	0.23
**8**	DRB3*02:02:01i	22.92%	27.59%	19.77%	1.45 (3.06)	0.32
**9**	DQA1*01:02:01G	7.64%	5.17%	9.30%	0.50 (1.94)	0.35
**10**	B*07:02:01G	11.81%	8.62%	13.95%	0.62 (1.67)	0.37
**11**	C*07:01:01G	27.78%	24.14%	30.23%	0.69 (1.57)	0.38
**12**	DQB1*03:01:01i	9.72%	12.07%	8.14%	1.64 (5.40)	0.41
**13**	DPB1*01:01:01G	9.72%	12.07%	8.14%	1.52 (4.68)	0.45
**14**	DRB4*01:01:01i	9.03%	6.90%	10.47%	0.69 (1.99)	0.51
**15**	DRB1*01:01:01i	16.67%	18.97%	15.12%	1.36 (3.50)	0.52
**16**	A*11:01:01G	5.56%	6.90%	4.65%	1.56 (7.15)	0.55
**17**	A*03:01:01G	16.67%	18.97%	15.12%	1.28 (3.03)	0.56
**18**	B*08:01:01G	24.31%	22.41%	25.58%	0.78 (1.99)	0.60
**19**	B*51:01:01G	9.03%	10.34%	8.14%	1.34 (4.54)	0.63
**20**	A*01:01:01G	24.31%	22.41%	25.58%	0.83 (1.87)	0.65
**21**	DQA1*03:01:01G	6.25%	5.17%	6.98%	0.71 (2.96)	0.65
**22**	B*35:01:01G	7.64%	8.62%	6.98%	1.28 (4.73)	0.70
**23**	C*05:01:01G	9.72%	8.62%	10.47%	0.82 (2.44)	0.72
**24**	DRB3*01:01:02i	32.64%	31.03%	33.72%	0.87 (1.85)	0.72
**25**	B*44:02:01G	7.64%	6.90%	8.14%	0.85 (2.79)	0.79
**26**	DPB1*02:01:02G	14.58%	15.52%	13.95%	1.13 (2.82)	0.80
**27**	DQA1*01:01:01G	21.53%	20.69%	22.09%	0.90 (2.22)	0.82
**28**	DRB1*13:01:01i	13.19%	13.79%	12.79%	1.11 (3.20)	0.85
**29**	DRB5*01:01:01i	5.56%	5.17%	5.81%	0.88 (3.89)	0.87
**30**	B*15:01:01G	5.56%	5.17%	5.81%	0.88 (3.89)	0.87
**31**	DQA1*01:03:01G	16.67%	17.24%	16.28%	1.08 (2.76)	0.87
**32**	DPB1*04:01:01G	36.81%	36.21%	37.21%	0.95 (2.03)	0.89
**33**	DQB1*05:01:01i	20.83%	20.69%	20.93%	0.98 (2.43)	0.97
**34**	DQB1*06:03:01i	13.89%	13.79%	13.95%	0.98 (2.80)	0.98
**35**	DQB1*02:01:01i	36.11%	36.21%	36.05%	1.01 (2.61)	0.98
**36**	C*03:03:01G	6.94%	6.90%	6.98%	0.99 (3.81)	0.98

### 3.9 Investigating whether T-LGL expansion affects anti-cN1A autoantibody production in IBM

Within this cohort of 85 IBM patients, we detected the presence of anti-cN1A in 29 patients, which corresponds to a prevalence of 34% (**Supplementary Figure 5K**). The presence of these autoantibodies was detected in 31.4% (16 out of 51 patients) of the T-LGL_LOW_ patient group, and in 38.2% (13 out of 34 patients) of the T-LGL_HIGH_ patient group. The measured trend for increased anti-cN1A seropositivity in patients of the T-LGL_HIGH_ compared to the T-LGL_LOW_ group was not statistically significant (OR=1.354, P=0.513). These results suggest that T-LGL expansion does not significantly promote the presence of anti-cN1A in IBM.

### 3.10 Clinical correlates of T-LGL expansions in IBM

Next, we examined whether the expansion of T-LGLs had clinical implications in IBM. First, we investigated whether any form of cytopenias (neutropenia, thrombocytopenia, or anemia) had been identified among IBM patients with expanded CD8^+^ T-LGLs and found that none had received such diagnosis. However, recurrent infections including mouth ulcers and chronic urinary tract infections were reported in 2/34 (5.8%) of T-LGL_HIGH_ patients. Of note, 23 IBM patients (11 T-LGL_HIGH_ and 12 T-LGL_LOW_) had autoimmune comorbidities, including Sjogren’s syndrome, rheumatoid arthritis, and coeliac disease (**Supplementary Table 1**).

**Figure 9:**
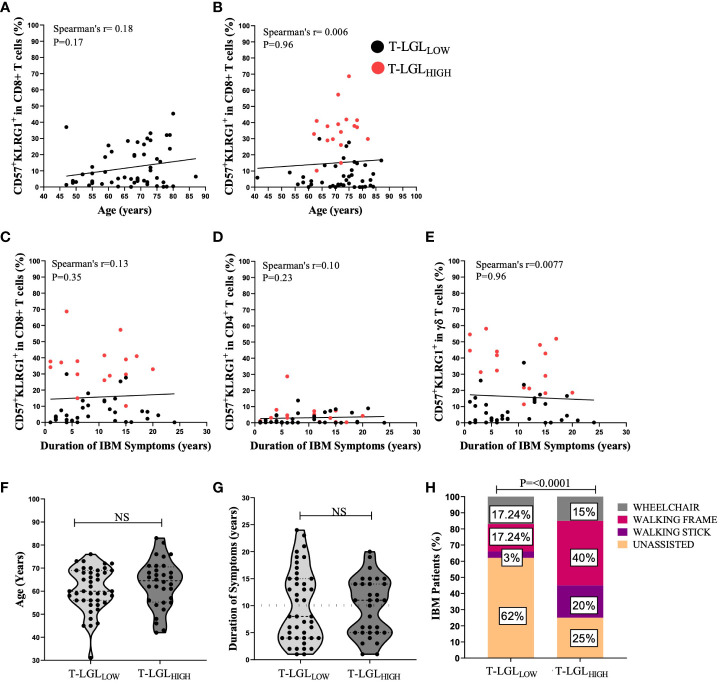
Clinical correlations of T-LGL expansions within IBM patients. **(A, B)** Spearman’s correlation between the proportion of CD8^+^CD57^+^KLRG1^+^ versus age in H.C (n=54) and IBM (n=59) respectively. **(C–E)** Spearman’s correlation shows the duration of IBM duration of symptoms (years) *versus* the proportion of CD8^+^CD57^+^KLRG1^+^ and CD4^+^CD57^+^KLRG1^+^ and γδ CD57^+^KLRG1^+^ respectively (n=52). **(F)** Difference between the age at onset (years) in IBM T-LGL_LOW_ patients (n=43) and IBM T-LGL_HIGH_ patients (n=32). Statistical analysis was performed using the two-tailed Mann-Whitney test for non-parametric data. **(G)** Difference between the duration of IBM symptoms (years) between IBM T-LGL_LOW_ (n=43) and IBM T-LGL_HIGH_ (n=32). Statistical analysis was performed using the two-tailed Mann-Whitney test for non-parametric data. **(H)** Graphical Representation shows the proportion of IBM patients and the various mobility aids used among IBM T-LGL_LOW_ patients (n=29) and IBM T-LGL_HIGH_ patients (n=20). Statistical analysis was performed using Pearson’s Chi-Square analysis. The P-values are indicated; NS, Not Significant.

Elevated frequency of T cells with a highly differentiated phenotype, such as CD57 and KLRG1 upregulation, have been reported to be associated with ageing (49, 50). However, we did not find any significant correlation between the proportion of CD57^+^KLRG1^+^ cells in CD8^+^ T cells with age in HC (Spearman’s r=0.1891, P=0.17, **Figure 9A**) or IBM cases (Spearman’s r=0.006, P*=*0.96, **Figure 9B**) Additionally, chronic antigenic stimulation has been found to result in T cell acquisition of a late-stage cellular differentiation phenotype featuring CD57 and KLRG1 upregulation (35, 51). We found that the IBM disease duration was not correlated with the proportion of CD57^+^KLRG1^+^ in any of the CD8^+^ (Spearman’s r=0.13, P=0.35*)*, CD4^+^ (Spearman’s r=0.10, P=0.23*)* or γδ T cells subsets (Spearman’s r= 0.0077, P=0.96; **Figures 9C–E** respectively). In addition, we found no significant differences in age at IBM disease onset in the T-LGL_HIGH_ and T-LGL_LOW_ IBM patient groups (median= 64.5 years, and 60 years respectively, P=0.3801, **Figure 9F**). Similarly, we found no differences in the duration of IBM symptoms between T-LGL_HIGH_ and T-LGL_LOW_ (11 years and 8 years respectively, P=0.91; **Figure 9G**). Interestingly, the distribution of both T-LGL_HIGH_ and T-LGL_LOW_ patients clustered into two distinct subgroups which can be demarcated by approximately the 10-year mark (**Figure 9G** dotted line). However, comparison of the patient repartition within these 10-year subgroups did not reveal any significant differences.

Additionally, we examined if the presence of T-LGL may contribute to IBM disease severity. We compared 29 T-LGL_LOW_ and 20 T-LGL_HIGH_ IBM patients based on their need for mobility aids (walking stick, walking frame, and wheelchair). Among IBM patients, 75% of the T-LGL_HIGH_ required mobility aids, ranging from a walking stick to a wheelchair, compared to only 38% of those with a T-LGL_LOW_ (P<0.0001; **Figure 9H**).

Overall, our results indicate that neither age at onset nor duration of IBM correlates with the expansion of CD8^+^CD57^+^KLRG1^+^ T-LGLs or with the proportional changes of CD57^+^ KLRG1^+^ in CD4^+^ and γδ T cells. However, patients who possessed high frequencies of T-LGL were more likely to require mobility aids, implying greater disease severity.

## 4 Discussion

IBM is a progressive inflammatory disease featuring skeletal muscle infiltration by highly differentiated CD8^+^ T cells (3). Although the exact etiology of IBM is still unknown, current research strongly supports a pathogenic role played by the autoimmune response (52). Thus, there is an ever-present need to better understand and characterize the immune changes within this disease. An association between IBM and T-LGL leukemia was previously reported in 58% of patients (13). In our study, we found that the frequency of CD8^+^CD57^+^ T-LGLs in total lymphocytes was elevated in the peripheral blood in 40% of an Australian cohort of IBM patients. However, only two of the T-LGL_HIGH_ patients in our study had T-LGL counts above the 0.5x10^9^/L threshold that is typically accepted for a diagnosis of T-LGL leukemia (16, 17) suggesting that most of the IBM patients in our cohort demonstrated only a mild expansion of these cells. The modest increase in the number of T-LGLs and the absence of associated cytopenias strongly suggest that this expanded T-LGL population may fall under the “reactive” classification of T-LGL lymphoproliferative disorders. Furthermore, the longitudinal examination of 14 IBM T-LGL_HIGH_ patients who were followed over a period of 7-32 months revealed a persistent and proportional increase of T-LGLs suggesting that stable expansions of these cells were not exceptional. However, in contrast to an earlier report (52), we did not assess TCR clonality in this study and therefore could not confirm whether this expansion of T-LGLs was reactive or leukemic.

The distinction between T-LGLL and reactive T-LGL expansions still presents many challenges and is often unclear, mostly due to the absence of consistent methodologies that have been applied to assess T cell clonality. Clonal analysis of hematological malignancies including T-LGLL is routinely assessed by polymerase chain reaction (PCR) for T-cell receptor gamma (*TCRG*) or TCR-beta (*TCRB*) gene rearrangements or TCRβ repertoire analysis using monoclonal antibodies and flow cytometry (16). The incidence of malignant T-LGLs among patients exhibiting clonal T cell expansion may be overestimated using these methods, as clonal expansion does not always imply malignancy. For instance, clonal expansion of T and B cells is a normal physiological response that follows initial antigen recognition (53). Additionally, the age-related contraction of the T cell repertoire may see the predominance of a limited number of clones especially those specific against lifelong viruses (54, 55). Thus, the classification of T-LGL leukemia as a T cell malignancy has generated some controversy, given the malignant nature of clonal expansions of T-LGLs generally follows an indolent course. Rather, these “neoplastic” T-LGLs may represent an exaggerated T cell response that have escaped normal homeostatic control. To date, few studies investigating the T-LGL TCR repertoire using high-throughput methods such as next generation sequencing have been performed (22, 56, 57). In this study, we did not assess T cell clonality, however, continuing efforts are underway to characterize the TCR sequences of T-LGLs in IBM using these in-depth sequencing technologies.

Cytopenias, such as anemia, neutropenia and thrombocytopenia are frequent complications seen in 40-60% of T-LGL leukemia cases owing to the production of anti-granulocyte antibodies or high circulating concentration of soluble FAS-ligand (14, 48, 58). In our cohort, none of the T-LGL_HIGH_ patients presented a diagnosis of a cytopenia according to standard laboratory testing, although we did note two patients, one with a history of chronic urinary tract infection (UTI) and the other with chronic mouth ulcers that are two conditions sometimes attributed to neutropenia (59). Also, several of the patients presented autoimmune co-morbidities including rheumatoid arthritis or Sjogren’s syndrome that have been documented as associated with T-LGLL (14, 60, 61). However, the incidence of these autoimmune co-morbidities was similar in both T-LGL_HIGH_ and T-LGL_LOW_ IBM patients, suggesting that a causal relationship is unlikely. We elected not to exclude patients with autoimmune co-morbidities from our studies for two key reasons: firstly our cohort consisted predominantly of elderly individuals (73 years on average) and the prevalence of co-morbidities within this age group is prominent and may therefore be relevant to the IBM-affected population in general, and secondly, our findings emphasize that T-LGL lymphoproliferations are prevalent in individuals afflicted by autoimmune disorders and that they may not simply arise in the context of a single autoimmune disease.

Within the expanded T-LGL population, we observed a highly differentiated immunophenotype characterized by increased CD57 combined with CD5 downregulation and an upregulation of natural killer receptors (NKRs) including CD94, KLRG1 and less consistently CD56. Expression changes of most of these molecules on CD8^+^ T cells was even more exacerbated in muscle tissues compared to blood, suggesting a role of the local inflammatory environment in this differentiation process. Furthermore, we found that similar phenotypical changes (most notably CD57 with co-expression of KLRG1 and a downregulation of CD5) extended to the CD4^+^ T cells and γδ T cells. Similarly, cases of CD4^+^ and γδ T cell T-LGL leukemia have been described previously, although their reported prevalence was lower than for CD8^+^ T-LGL leukemia (16, 19, 30). To our knowledge, our study represents the first instance of simultaneously investigating these phenotypical changes across the CD8^+^, CD4^+^ and γδ T cell landscape in IBM.

The immunophenotype of the expanded T-LGLs raises some interesting scenarios about their regulation and activity. For instance, CD5 is a transmembrane glycoprotein forming a receptor expressed on T cells that negatively regulates the TCR activity (62). Therefore, downregulation of CD5 as seen in T-LGL, could lead to increased responsiveness. Similarly, we found that the lectin-like NKR CD94 was moderately upregulated on the CD8^+^ and γδ T-LGL, while CD4^+^ T cells remained negative. CD94 forms an heterodimeric receptor by associating with NKG2A that is inhibitory or with NKG2C, -E or -H that are activating and regulate functions such as cytotoxicity and cytokine production (63). Therefore, the functional significance of CD94 upregulation will remain unclear until the associated receptor is characterized and its regulatory function on TCR-dependent and independent activation pathways is demonstrated. Nonetheless, a study has reported that the activating receptor NKG2C/E forms a dimer with CD94 on T-LGLs (64). Alternatively, CD94 pairing with NKG2A and forming an inhibitory receptor has been implicated in T-LGL associated-pathology, which could offer prognostic value. For instance, two independent studies, one on CD8^+^ T-LGLL (65) and the other on γδ T-LGLL (66), found T cells expressing NKG2A in patients with a “milder” disease burden; a role was attributed to NKG2A inhibitory effect on cytokine release. Thus, CD94-associated inhibitory receptors might contribute to preventing T-LGL cell putative autoreactivity. Clarifying which of the CD94 -NKG2A or C heterodimers are expressed on T-LGL populations may reveal the underlying regulatory mechanisms and their functional significance in IBM along with other autoimmune disorders and ultimately lead to the identification of useful prognostic biomarkers and the development of novel therapeutic interventions.

Co-expression of CD57 and KLRG1 on CD8^+^ T cells have previously been denoted as markers of T cell senescence (51, 67, 68). This dysfunctional state is characterized by short telomeres and inability to undergo clonal expansion after stimulation (67). However, in contrast to exhausted cells, senescent cells retain the capacity to produce high levels of pro-inflammatory cytokines and cytotoxic granules (67, 68). KLRG1 is an inhibitory receptor that hinders proliferation upon engaging its ligands, E and N cadherins (51). Recent studies reported elevated KLRG1 expression on peripheral blood CD8^+^ and CD4^+^ T cells in IBM (36). Our study reaffirms these observations, although we noted the expression of KLRG1 is greater in IBM T-LGL_HIGH_ than in T-LGL_LOW_ patients and HC. Additionally, we noted a higher proportion of CD57^+^KLRG1^+^ within the CD4^+^ and γδ T cell subsets in T-LGL_HIGH_ individuals. The expression of KLRG1 in CD4^+^ T cells in IBM patients’ blood and muscle tissue has been reported only recently (36, 69). Goyal et al. observed that KLRG1^+^ CD4^+^ T cells had down-regulated CD28 while producing IFN-γ and perforin, indicating inflammatory and cytotoxic functions (36). Cytotoxic CD4^+^ T cells displaying similar features have been described previously in chronic viral infections, cancer and autoimmunity, suggesting that these changes likely result from persistent antigenic stimulation (70). Our analysis of the CD4^+^ subset showed an abundance of pro-inflammatory (IFN-γ) and cytotoxic (perforin) content which was more pronounced in the T-LGL_HIGH_ IBM patients. Nonetheless, cytotoxic CD4^+^ T cells may be contributing to the overall immune dysregulation within this subgroup of IBM patients.

The expression of senescence markers on T-LGL brings into question their proliferative capability. We evaluated our T-LGL population *in vivo* using the presence of intranuclear Ki67 as a reliable marker of proliferation. In agreement with findings from a previous study by Greenberg and coworkers that muscle-invading KLRG1^+^ T cells are minimally or non-proliferative (3), our study shows that a small fraction of CD8^+^CD57^+^KLRG1^+^ T-LGLs retained a proliferative activity, even though Ki67 expression was reduced compared to their less differentiated CD8^+^CD57^-^KLRG1^-^ counterparts. We propose that T-LGL in IBM likely arise from a less differentiated memory cell population. Although their proliferation is significantly limited, they are still capable to some extent of proliferating which contributes to their persistence over prolonged periods of time.

The increased expression of KLRG1 in γδ T cell subsets detected in T-LGL_HIGH_ IBM patients opens new research directions. γδ T cells represent a relatively small subpopulation of T cells in blood; they recognize microbial and stress-induced phosphorylated non-peptidic antigens and share features of both innate and adaptive immune cells (71). In addition, γδ T cells are tightly regulated by activating and inhibitory NKRs (72, 73). The expression of KLRG1 on γδ T cells has not received as much attention as for their αβ counterparts, thus their functional significance in IBM should be more closely investigated in future studies.

The HLA is a key genetic determinant that may predispose to autoimmune diseases. In IBM, HLA associations with alleles: more particularly HLA-DRB1*03:01 and alleles of the 8.1 MHC ancestral haplotype strengthen the argument in favor of IBM’s autoimmune etiology (11). Similarly, carriage of the HLA-DR4 allele frequency was exceedingly present in T-LGLL patients with RA (74, 75). We assessed potential HLA class I and class II allele associations in T-LGL_HIGH_ and T-LGL_LOW_ patients, which may indicate a role for presentation of antigen to CD8^+^ or CD4^+^ T cells, respectively. We found increased carriage frequency of the HLA-C*14:02:01 allele within T-LGL_HIGH_ patients compared to the T-LGL_LOW_ group, indicating that this HLA class I allele may represent a risk factor, possibly through preferential presentation of IBM-related antigens. Future analysis of the specificity of expanded T-LGL clones will provide a more precise understanding of these mechanisms. However, the limited representation of this allele that only accounts for 12.1% of the T-LGL_HIGH_ patients indicates that it is dispensable and questions the extent of its role during T-LGL expansion. We propose additional investigation into this genetic association in a larger IBM cohort should be conducted to confirm this finding.

Ageing along with pathological chronic conditions such as viral infections, cancer and autoimmunity, where the T cells are exposed to persistent antigenic stimulation, is thought to drive the increased frequency of cells displaying markers of senescence including CD57 and KLRG1 (35, 51). Although we endeavored to compare the patient group to an age-matched healthy control group, and recruited donors among the patients’ spouses; the median age is 5 years less than the patient group (68 vs 73 year old) and this difference is statistically significant (P=0.0018). However, this age gap is unlikely to be clinically relevant considering that the occurrence of T-LGL has been associated with ageing, with a reported median age of 66.5 at diagnosis in a study that collected data covering 28% of the US population (76). The control group (68 year old) is recognized as older-adults according to the CDC definition (>65 year old), and therefore the immune system alterations associated with normal ageing provide a valid reference against the IBM group. We did not find any significant correlation between IBM patients’ age and/or the duration of disease with the CD57^+^KLRG1^+^ phenotype in the CD8^+^, CD4^+^ and γδ T cell subsets. A recent study investigated similar parameters and reported a moderate correlation between CD8^+^KLRG1^+^ TEMRA cells and disease duration (36). A limitation about accurately recording disease duration relies on the ability of patients to recall when symptoms first emerged; this introduces recollection bias especially considering that symptoms often arise years before a diagnosis of IBM is confirmed. However, just as the development of autoimmunity is a multifactorial, complex process involving environmental, genetic, transcriptional, and epigenetic components, the development of T-LGL lymphoproliferative disorders including T-LGLL is also likely to be multifactorial, and ageing or chronic antigen stimulation alone may not be sufficient to promote the expansion of this disorder.

Greenberg *and coworkers* reported that T-LGL expansions resulted in a more severe clinical phenotype indicated by a greater decline in muscle strength (13). In this study, we used patients’ reliance on mobility aids as a surrogate measure of disease severity. Our findings that T-LGL_HIGH_ patients experienced a more severely reduced mobility compared to T-LGL_LOW_ patients confirmed the conclusions of this previous research. Considering that the proportion of T-LGLs was not correlated to ageing or disease duration, we can exclude that the increased reliance on mobility aids was not due to the normal ageing or progression of the disease, and rather propose that it may be causally linked to the expansion of this late differentiated T-LGL cell population.

Even though it remains difficult to conclude whether the T-LGL expansion that we observed in this cohort of IBM patients is representative of T-LGL leukemia, given the relatively low T-LGL numbers and the absence of associated cytopenias, we propose that in the majority of patients these T cell expansions fall into the category of “reactive” T-LGL lymphoproliferative disease (17) and are more likely to be secondary, rather than a driver of IBM. We report a group of IBM patients who display an elevated proportion of CD8^+^CD57^+^ cells with a senescent-like profile associated with upregulation of inhibitory NK cell receptors. These aberrant changes across the CD8^+^, CD4^+^ and γδ T cell landscape suggest altered TCR-dependent responsiveness, increased inflammatory and cytotoxic features, and reduced proliferative capabilities. These changes likely culminate in exacerbated immune dysregulation and increased disease burden occurring within this subgroup of IBM patients. This immunophenotype may represent an effective way to stratify IBM patients and may assist in providing a prognostic tool and in identifying suitable candidates for future targeted therapies.

## Data availability statement

The data presented for the HLA allele sequences are deposited in the NCBI Sequence read Archive (SRA) repository; the accession number is PRJNA945219.

## Ethics statement

The studies involving human participants were reviewed and approved by Murdoch University HREC, protocol #2015/111. The patients/participants provided their written informed consent to participate in this study.

## Author contributions

Conceptualization: EM and JDC. Provided biological samples: KB, PJL, SP and MN. Performed experiments: EM, NS, AS, AC and JDC. Statistical analysis: EM, BK and JDC. Writing – original draft: EM and JDC. Reviewed and edited the manuscript: EM, AS, NS, BK, SP, PJL, AC, FLM, MN, and JDC. Figures: EM and JDC. Funding acquisition: MN and JDC. Supervision: MN and JDC. All authors contributed to the article and approved the submitted version.
