# Supplementary material for: Uncovering the significance of expanded CD8+ large granular lymphocytes in inclusion body myositis: Insights into T cell phenotype and functional alterations, and disease severity
Source: Front Immunol. 2023 Mar 30;14:1153789. doi: 10.3389/fimmu.2023.1153789 (PMC10098158; doi:10.3389/fimmu.2023.1153789)
Supplement: Supplementary file 1 [file DataSheet_1.pdf]

Supplementary Material

Uncovering the significance of expanded CD8+ Large Granular Lymphocytes in Inclusion Body Myositis: Insights into T cell phenotype and functional alterations, and disease severity.

E. McLeish[1]*, A. Sooda (Ph.D.)[1], N. Slater[1], B. Kachigunda (Ph.D.)[2] K. Beer[3], S. Paramalingam[4], P.J. Lamont[5], A. Chopra[1,6], Frank L. Mastaglia (MBBS, MD, FRACP, Ph.D.) [3], M. Needham (MBBS, FRACP, Ph.D.) [1, 3,7,8], J.D. Coudert (Ph.D.)[1,3,7*]

*Corresponding author: E.McLeish@iiid.murdoch.edu.au, jerome.coudert@murdoch.edu.au

This page is Intentionally left blank

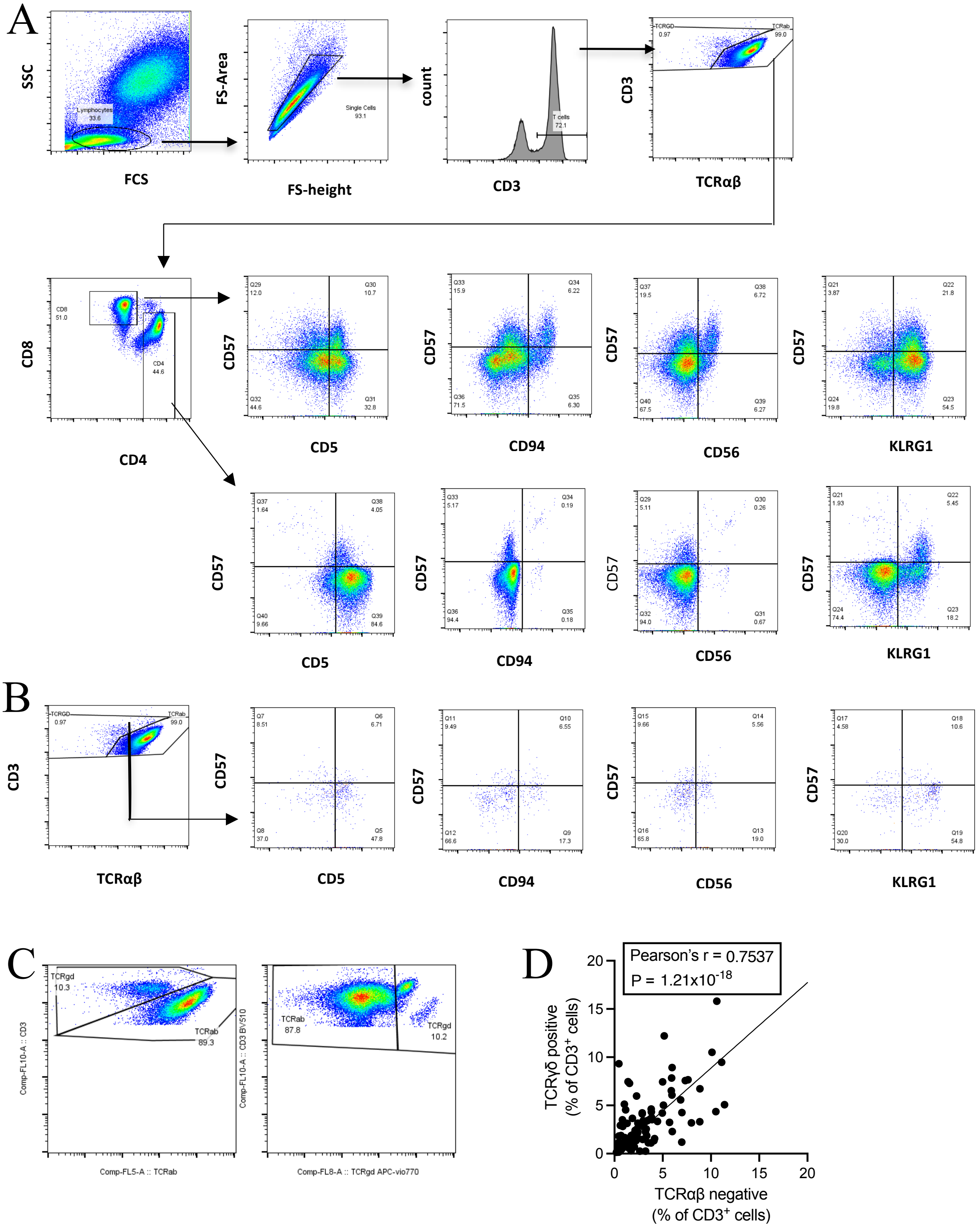

Supplementary Figure 1: Representative flow cytometry gating strategy for differentiation markers on αβ (**A**) and γδ (gated on αβ-negative fraction of $CD3^{+}$) (**B**) T cells. **C.** Representative illustration of γδ T cell population visualized by a negative gating strategy (gated on αβ-negative fraction of $CD3^{+}$; left dot plot) and by a positive gating strategy (gated on γδ-positive fraction of $CD3^{+}$ using an alternate antibody panel* on the same blood sample; right dot plot). **D.** Correlation analysis between the percentage of TCRγδ in $CD3^{+}$ T cells measured using the negative and the positive γδ-gating strategies. Ninety-five paired values measured from IBM and HC samples were compared using the Pearson's correlation test.

* CD3-BV510 (clone UCHT1, BD Bioscience, NJ, USA) , TCR-γδ–APC-Vio770 (clone REA591, Milteniy Biotec, Maquarie Park, NSW, Australia).

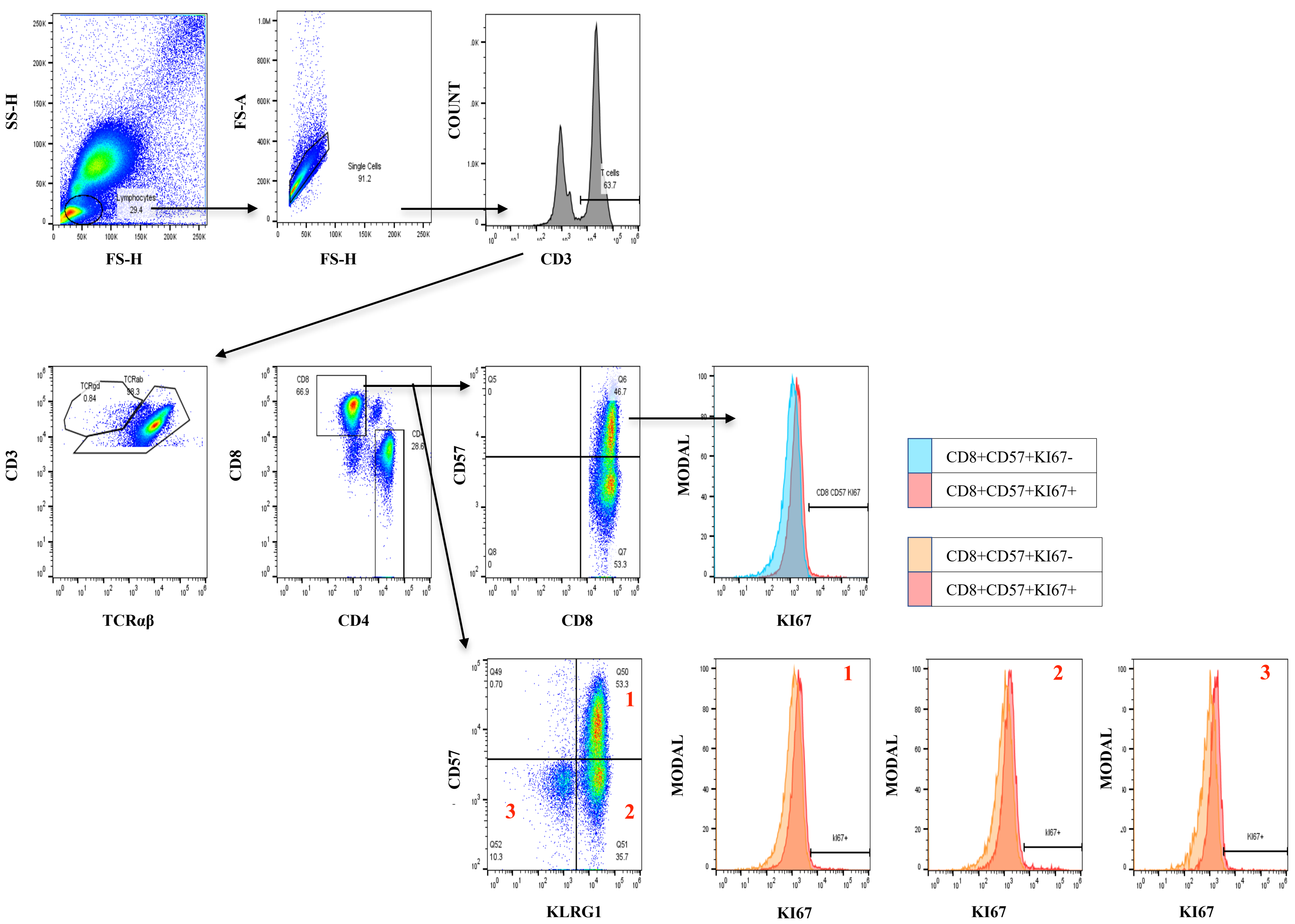

Supplementary Figure 2: Representative Flow cytometry gating strategy for KI67 expression in T cells.

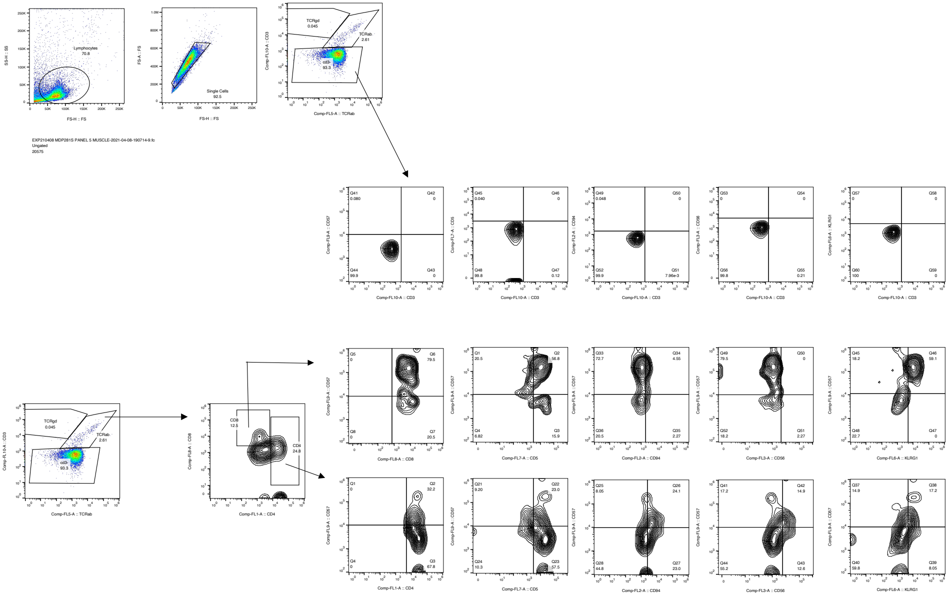

Supplementary Figure 3: Representative Flow cytometry gating strategy for muscle-isolated lymphocytes.

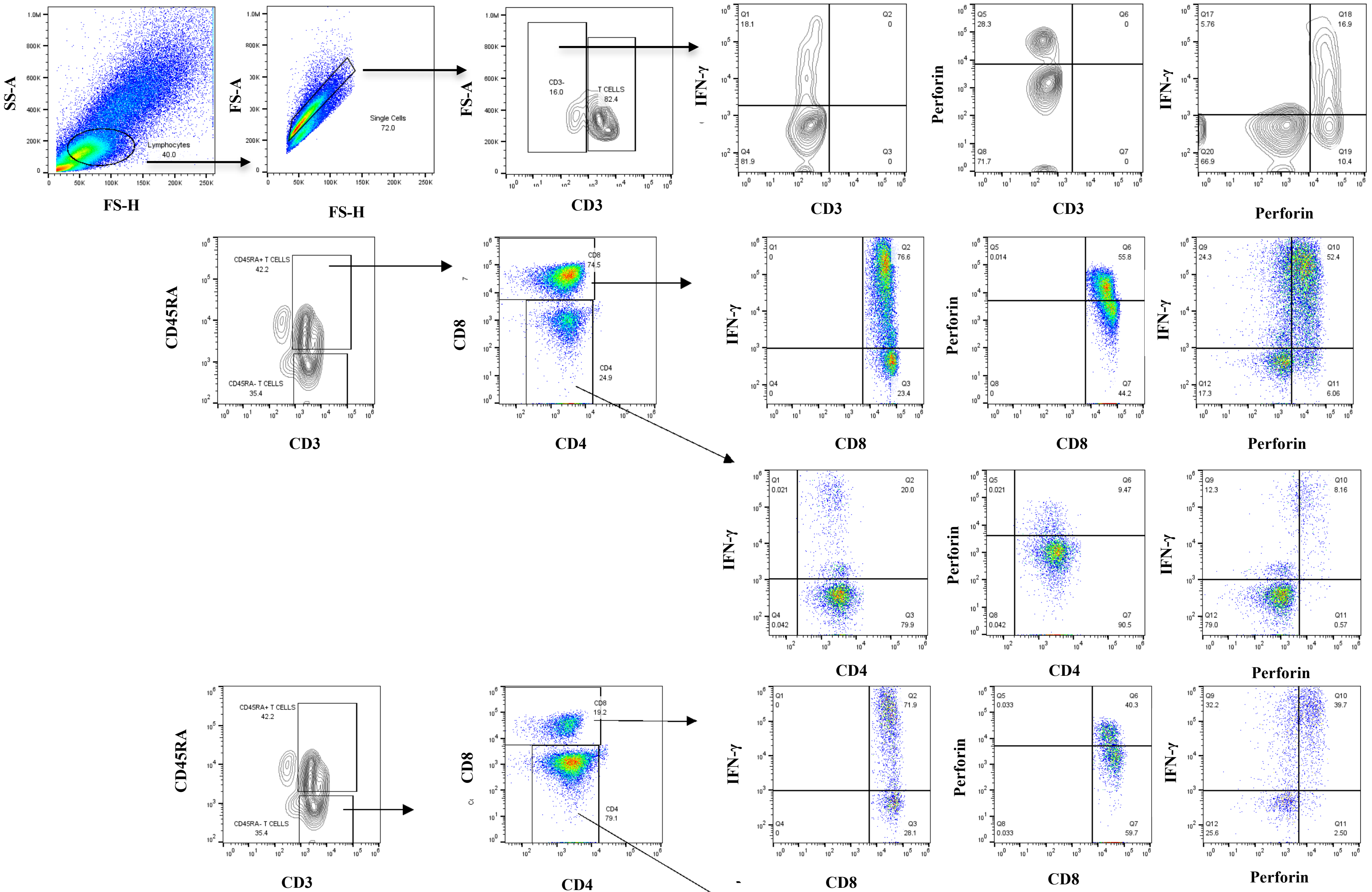

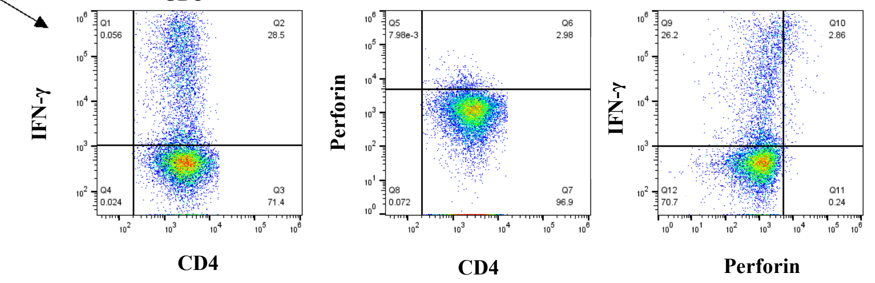

Supplementary Figure 4: Representative Flow cytometry gating strategy for intracellular of IFN-γ and perforin in T cells.

Supplementary Table 1: IBM Patients Clinical Data

Subject	*Age*	*Gender*	*Ethnicity*	*T-LGL status*	*Persistent/Transient*	*Years since onset*	*% T-LGL in Lymphocytes*	*CD4/ CD8 ratio*	*Co-morbidities*
1	67	M	CAUCASIAN	HIGH	Persistent	5	7.13%	1.14	
2	68	F	CAUCASIAN	HIGH	Persistent	5	19.68%	1.39	
3	79	F	CAUCASIAN	HIGH	NA	NA	6.38%	1.26	IgA Lambda, Multiple Myeloma, Breast cancer
4	57	M	CAUCASIAN	HIGH	Persistent	5	44.27%	0.19	
5	76	M	CAUCASIAN	HIGH	NA	11	13.04%	1.35	chronic mouth ulcers, Type 2 Diabetes
6	80	M	CAUCASIAN	HIGH	Persistent	11	6.54%	2.47	
7	58	F	CAUCASIAN	HIGH	NA	3	23.51%	0.54	
8	96	M	CAUCASIAN	HIGH	persistent	15	16.27%	0.97	
9	62	F	CAUCASIAN	HIGH	persistent	19	7.41%	2.85	
10	88	M	CAUCASIAN	HIGH	Persistent	5	7.01%	2.93	
11	74	F	CAUCASIAN	HIGH	Unknown	12	5.98%	2.51	
12	76	F	CAUCASIAN	HIGH	NA	8	3.36%	1.79	
13	81	F	CAUCASIAN	HIGH	Persistent	14	23.91%	0.54	
14	75	F	CAUCASIAN	HIGH	NA	14	4.25%	1.20	
15	86	F	CAUCASIAN	HIGH	NA	10	11.48%	0.79	
16	78	F	CAUCASIAN	HIGH	Persistent	14	7.96%	1.83	
17	73	F	CAUCASIAN	HIGH	Persistent	5	6.71%	1.52	Rheumatoid Arthritis, Sjogren's disease
18	71	F	ASIAN	HIGH	Persistent	14	22.94%	0.58	Sjogren's disease
19	67	M	CAUCASIAN	HIGH	Unknown	12	8.34%	0.62	Haematuria, JAK-2 Mutation,
20	67	M	CAUCASIAN	HIGH	Persistent	1	10.46%	0.73	Castleman's Disease, Thyroid cancer, Type 2 diabetes
21	63	F	NA	HIGH	NA	17	10.70%	1.57	
22	74	M	CAUCASIAN	HIGH	NA	NA	8.83%	1.66	
23	72	M	CAUCASIAN	HIGH	NA	1	12.65%	0.91	Hypothyroid disease, past bowel cancer, severe lung infection

24	63	M	CAUCASIAN	HIGH	NA	15	10.28%	0.58	
25	71	M	CAUCASIAN	HIGH	Persistent	15	5.50%	1.77	
26	62	M	CAUCASIAN	HIGH	NA	20	8.68%	0.91	
27	82	M	CAUCASIAN	HIGH	NA	6	6.73%	0.46	
28	77	M	CAUCASIAN	HIGH	NA	6	10.84%	0.85	Graves Disease, Barrett's esophagus, type 1 diabetes
29	78	F	NA	HIGH	NA	3	16.59%	0.57	Rheumatoid Arthritis, Type 2 Diabetes, Asthma, Dyslipidemia
30	69	M	CAUCASIAN	HIGH	NA	15	9.41%	0.99	
31	72	F	NA	HIGH	Persistent	11	9.94%	1.92	
32	78	M	NA	HIGH	NA	11	7.90%	2.33	
33	75	M	NA	HIGH	NA	4	25.96%	0.79	
34	72	M	CAUCASIAN	HIGH	NA	6	4.10%	1.46	
35	49	M	CAUCASIAN	LOW	unchanged	4	3.24%	2.82	
36	68	F	CAUCASIAN	LOW	CD8 Increasing	17	1.48%	3.02	myasthenia gravis, Fatigue Like symptoms
37	71	M	CAUCASIAN	LOW	unchanged	23	0.88%	1.81	
38	75	F	CAUCASIAN	LOW	CD8 Increasing	NA	2.16%	1.45	
39	46	M	NA	LOW	unchanged	15	5.59%	0.01	
40	78	M	CAUCASIAN	LOW	NA	20	6.94%	2.44	Psoriasis
41	62	F	CAUCASIAN	LOW	unchanged	4	4.59%	2.22	
42	81	M	CAUCASIAN	LOW	NA	14	0.28%	2.21	
43	62	M	CAUCASIAN	LOW	NA	NA	0.55%	9.02	
44	80	M	1/2 CAUCASIAN 1/2 ASIAN	LOW	unchanged	24	1.23%	2.65	Hyperthyroid
45	76	M	CAUCASIAN	LOW	NA	3	3.50%	2.10	Asthma
46	83	F	CAUCASIAN	LOW	CD8 Increasing	13	0.65%	7.02	
47	72	F	CAUCASIAN	LOW	NA	4	0.14%	4.46	
48	81	M	CAUCASIAN	LOW	Progressing	11	0.45%	27.86	Coeliac disease
49	74	M	CAUCASIAN	LOW	unchanged	14	1.56%	5.22	
50	75	F	CAUCASIAN	LOW	unchanged	19	0.48%	5.72	Melanoma, Hypothyroid
51	77	F	NA	LOW	NA	NA	0.89%	10.08	Muscular dystrophy, Rheumatoid Arthritis
52	69	M	CAUCASIAN	LOW	unchanged	5	0.51%	2.34	

53	73	F	CAUCASIAN	LOW	NA	8	3.60%	1.42	
54	76	M	CAUCASIAN	LOW	unchanged	8	3.71%	1.50	
55	60	M	CAUCASIAN	LOW	NA	6	0.21%	3.59	
56	66	F	CAUCASIAN	LOW	NA	7	3.39%	1.77	
57	84	F	CAUCASIAN	LOW	NA	12	5.01%	1.61	
58	74	M	CAUCASIAN	LOW	NA	4	1.90%	2.17	
59	53	F	CAUCASIAN	LOW	Progressing	7	2.68%	2.63	Coeliac disease
60	71	F	CAUCASIAN	LOW	NA	2	0.51%	1.97	
61	70	M	UNKNOWN	LOW	unchanged	4	0.17%	1.86	Type 2 diabetes
62	65	M	CAUCASIAN	LOW	NA	6	3.08%	1.89	
63	75	M	CAUCASIAN	LOW	Progressing	15	4.74%	3.99	Melanoma
64	81	M	CAUCASIAN	LOW	unchanged	NA	0.03%	8.90	
65	73	F	CAUCASIAN	LOW	NA	18	4.30%	1.53	
66	83	F	CAUCASIAN	LOW	NA	21	2.67%	2.29	
67	77	M	CAUCASIAN	LOW	unchanged	1	0.42%	1.00	Chronic Kidney Disease, Type 2 diabetes, melanoma
68	84	M	CAUCASIAN	LOW	NA	15	0.21%	4.39	
69	41	F	CAUCASIAN	LOW	NA	NA	0.30%	5.52	
70	79	F	CAUCASIAN	LOW	unchanged	6	0.21%	4.17	
71	68	F	CAUCASIAN	LOW	NA	16	0.39%	8.93	insulin resistant
72	56	M	CAUCASIAN	LOW	unchanged	2	1.28%	5.47	
73	61	F		LOW	NA	NA	1.76%	3.23	
74	75	M	CAUCASIAN	LOW	unchanged	19	0.22%	3.29	Type 1 diabetes
75	80	M	CAUCASIAN	LOW	NA	8	0.25%	9.50	
76	70	F	CAUCASIAN	LOW	NA	11	2.63%	3.32	
77	70	F	NA	LOW	NA	1	0.29%	4.90	
78	58	F	NA	LOW	NA	13	1.32%	5.40	
79	58	F	NA	LOW	NA	2	0.96%	4.10	
80	61	M	NA	LOW	NA	2	0.42%	3.51	
81	87	M	NA	LOW	NA	NA	4.50%	2.03	
82	78	F	CAUCASIAN	LOW	unchanged	13	2.13%	4.39	
83	73	M	NA	LOW	NA	5	0.23%	2.81	
84	64	M	NA	LOW	NA	4	6.39%	1.86	
85	71	M	NA	LOW	NA	NA	0.15%	2.29	

Subject	Age	Gender	Ethnicity	T-LGL status	Persistent/Transient	NOY	% T-LGL in Lymphocytes	CD4/CD8 ratio	Co-morbidities
1	59	F	-	-	-	-	2.59%	3.20	-
2	65	M	-	-	-	-	0.78%	2.42	-
3	55	M	-	-	-	-	0.71%	2.15	-
4	52	F	-	-	-	-	0.25%	4.15	-
5	69	M	-	-	-	-	0.58%	4.44	-
6	78	F	-	-	-	-	0.16%	2.57	-
7	72	M	-	-	-	-	0.53%	3.74	-
8	75	F	-	-	-	-	2.01%	1.34	-
9	72	F	-	-	-	-	5.73%	2.08	-
10	78	M	-	-	-	-	3.06%	5.24	-
11	73	M	-	-	-	-	6.18%	2.14	-
12	68	M	-	-	-	-	2.18%	0.30	-
13	55	F	-	-	-	-	0.18%	4.94	-
14	63	M	-	-	-	-	0.03%	13.90	-
15	76	M	-	-	-	-	1.21%	3.55	-
16	47	M	-	-	-	-	6.58%	2.31	-
17	49	F	-	-	-	-	0.73%	3.32	-
18	73	F	-	-	-	-	2.25%	1.37	-
19	73	F	-	-	-	-	0.31%	15.19	-
20	87	M	-	-	-	-	1.56%	3.47	-
21	59	F	-	-	-	-	4.67%	1.41	-
22	80	F	-	-	-	-	5.84%	3.30	-
23	80	M	-	-	-	-	0.79%	5.00	-
24	49	M	-	-	-	-	1.50%	2.09	-
25	77	M	-	-	-	-	0.59%	3.32	-
26	71	M	-	-	-	-	5.73%	1.30	-
27	59	F	-	-	-	-	1.16%	3.81	-

28	60	F	-	-	-	-	6.29%	1.77	-
29	79	M	-	-	-	-	6.45%	1.50	-
30	75	M	-	-	-	-	0.20%	2.78	-
31	73	F	-	-	-	-	5.16%	2.73	-
32	68	F	-	-	-	-	0.24%	5.17	-
33	58	F	-	-	-	-	0.70%	2.06	-
34	67	M	-	-	-	-	1.36%	1.31	-
35	47	F	-	-	-	-	1.05%	1	-
36	54	F	-	-	-	-	0.75%	2.38	-
37	69	F	-	-	-	-	0.10%	4.51	-
38	72	F	-	-	-	-	0.18%	1.64	-
39	67	M	-	-	-	-	0.00%	4.11	-
40	63	M	-	-	-	-	1.38%	1.51	-
41	50	F	-	-	-	-	0.19%	6.02	-
42	65	M	-	-	-	-	1.67%	1.13	-
43	66	M	-	-	-	-	4.62%	1.15	-
44	55	M	-	-	-	-	0.13%	1.86	-
45	55	M	-	-	-	-	0.33%	3.5	-
46	77	M	-	-	-	-	0.01%	3.86	-
47	61	F	-	-	-	-	0.10%	7.86	-
48	71	F	-	-	-	-	0.09%	3.08	-
49	75	F	-	-	-	-	0.68%	3.27	-
50	71	M	-	-	-	-	2.38%	2.70	-
51	68	F	-	-	-	-	2.28%	5.68	-
52	79	M	-	-	-	-	2.33%	3.04	-
53	55	F	-	-	-	-	3.21%	1.83	-
54	61	F	-	-	-	-	7.71%	1.55	-
55	69	F	-	-	-	-	0.70%	4.55	-

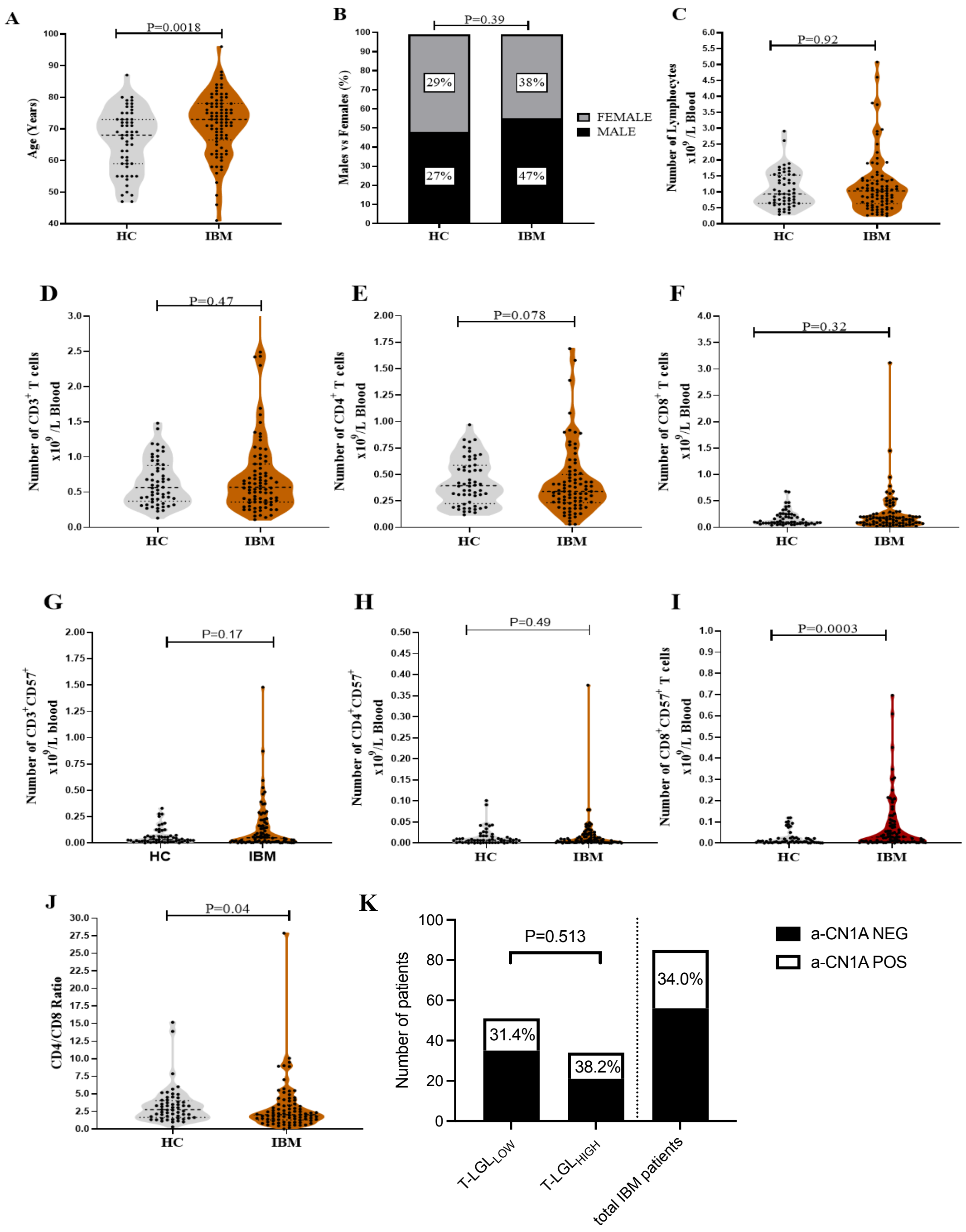

Supplementary Figure 5

Study cohort demographics and leukocyte counts and autoantibodies: 56 HC vs 85 IBM patients were analysed in this figures **A.** Age (years). statistical analysis was performed using Mann-Whitney test for non-parametric data. **B.** Number of Males vs Females. Statistical analysis was performed using two sided Fishers Exact tests. **C**. Total number of lymphocytes/L blood. statistical analysis was performed using Mann-Whitney test for non-parametric data. **D.** Number of $CD3^+$ T cells/ L blood. statistical analysis was performed using Mann-Whitney test for non-parametric data**. E.** Number of $CD4^+$ T cells/ L blood. Statistical analysis was performed using Mann-Whitney test for non-parametric data**. F.** Number of $CD8^+$ T cells/ L blood. Statistical analysis was performed using Mann-Whitney test

for non-parametric data**.** Statistical analysis was performed using Mann-Whitney test for non-parametric data**. G-I.** Number of $CD3^{+}CD57^{+}$, $CD8^{+}CD57^{+}$, $CD4^{+}CD57^{+}$ per litre of blood. Statistical analysis was performed using Mann-Whitney test for non-parametric data. **J.** CD4/CD8 ratio in IBM and HC donor group. **K.** The presence of anti-CN1A antibodies was assessed in the serum of n=51 T-LGL_{LOW} and n=34 LGL_{HIGH} IBM patients. The number of seronegative and seropositive patients in each group is shown. The values within the open bars indicate the percentage of seropositive patients within each group. Statistical analysis was performed using the two-sided Person's Chi-square test. The numbers of seropositive and seronegative patients in the whole IBM patient cohort (n=85) are shown as a reference.

Supplementary Table 2: Proportion of the relative Surface markers in $CD8^{+}$ $CD4^{+}$ and γδ T Cells between Healthy Controls and IBM patients with and without T-LGL expansions

		Healthy Controls N=56			IBM T-LGL$_{LOW}$ N=51			IBM T-LGL$_{HIGH}$ N=34			P-value
	Marker	Mean (%) + SD	Median (range)	95%CI ranges	Mean (%) + SD	Median (range)	95%CI	Mean (%) + SD	Median (range)	95%CI	Kruskal- Wallis ANOVA
CD8	CD57CD5DIM	3.25± 5.09	0.69 (0.0-18.90)	1.9- 4.6	2.171± 3.57	0.95 (0.0-16.6)	1.1- 3.1	23.21± 12.70	19.9 (7.7-67.80)	18.8- 27.6	<0.0001
	CD57CD94	0.97± 2.015	(0-11)	0.43-1.51	0.87± 1.62	0.26 (0-8.81)	0.41-1.33	8.74± 8.31	5.53 (0.80-33)	5.83-11.63	<0.0001
	CD57CD56	1.12± 2.14	0.23 (0-12.20)	0.54-1.70	0.74± 1.74	0.14 (0-9.9)	0.23-1.24	7.34± 6.77	5.0 (0.08-23.10)	5.0- 9.7	<0.0001
	CD57KLRG1*	11.94± 11.94	5.74 (0.05-45.3)	8.7-15.2	6.8± 7.89	3.73 (0-29.93)	4.30-9.22	35.82± 13.64	37.10 (10.2-69.70)	28.81-42.83	<0.0001
CD4	CD57CD5DIM	0.92± 1.8	0.145 (0.0-9.27)	0.44-1.4	0.50± 1.73	0.077 (0.0-11.60)	0.01-1.0	2.4± 3.25	1.51 (0.56-17.6)	1.22- 3.49	<0.0001
	CD57CD94	0.1342± 0.168	0.07 (0.0-0.87)	0.1-0.17	0.054± 0.084	0.03 (0.0-0.56)	0.03-0.08	0.35± 0.45	0.15 (0.0-2.09)	0.19- 0.50	<0.0001

	CD57CD56	0.34± 1.53	0.06 (0.0-11.90)	-0.06-0.75	0.08± 0.14	0.034 (0.0-0.69)	0.04-0.11	0.36± 0.65	0.185 (0.01-3.751)	0.13- 0.59	<0.0001
	CD57KLRG1*	2.1± 4.6	0.24 (0.0-23.1)	0.82-3.3	2.06± 3.19	0.36 (0.0-13.80)	1.06-3.05	4.915± 6.7	3.08 (0.24-28.70)	1.5- 8.35	0.0018
**TCRγδ**	CD57CD5DIM	9.4± 11.10	6.24 (0.0-63.7)	6.40-12.35	5.09± 5.3	3.56 (0.-25.10)	3.60-6.6	17.80 16.65	11.05 (0.49-58.40)	12-23.61	<0.0001
	CD57CD94	3.70± 6.7	1.24 (0.0-43.20)	1.91-5.5	2.1 3.10	0.52 (0.0-11.80)	1.22-2.97	12.3 15.27	6.5 (0.65-69.10)	7.01-17.67	<0.0001
	CD57CD56	3.70± 6.0	1.54 (0.0-27)	2.1-5.30	1.85 2.6	1.16 (0-11.60)	1.1-2.58	10.09 16.52	4.40 (0.00-83.30)	4.3- 15.85	0.0002
	CD57KLRG1*	9.7± 10.04	6.52 (0.0-39.30)	7.0-12.45	7.0 8.45	2.51 (0.37.10)	4.3- 9.6	35.21 14.75	36.40 (11.20-58.10)	27.63-42.80	<0.0001

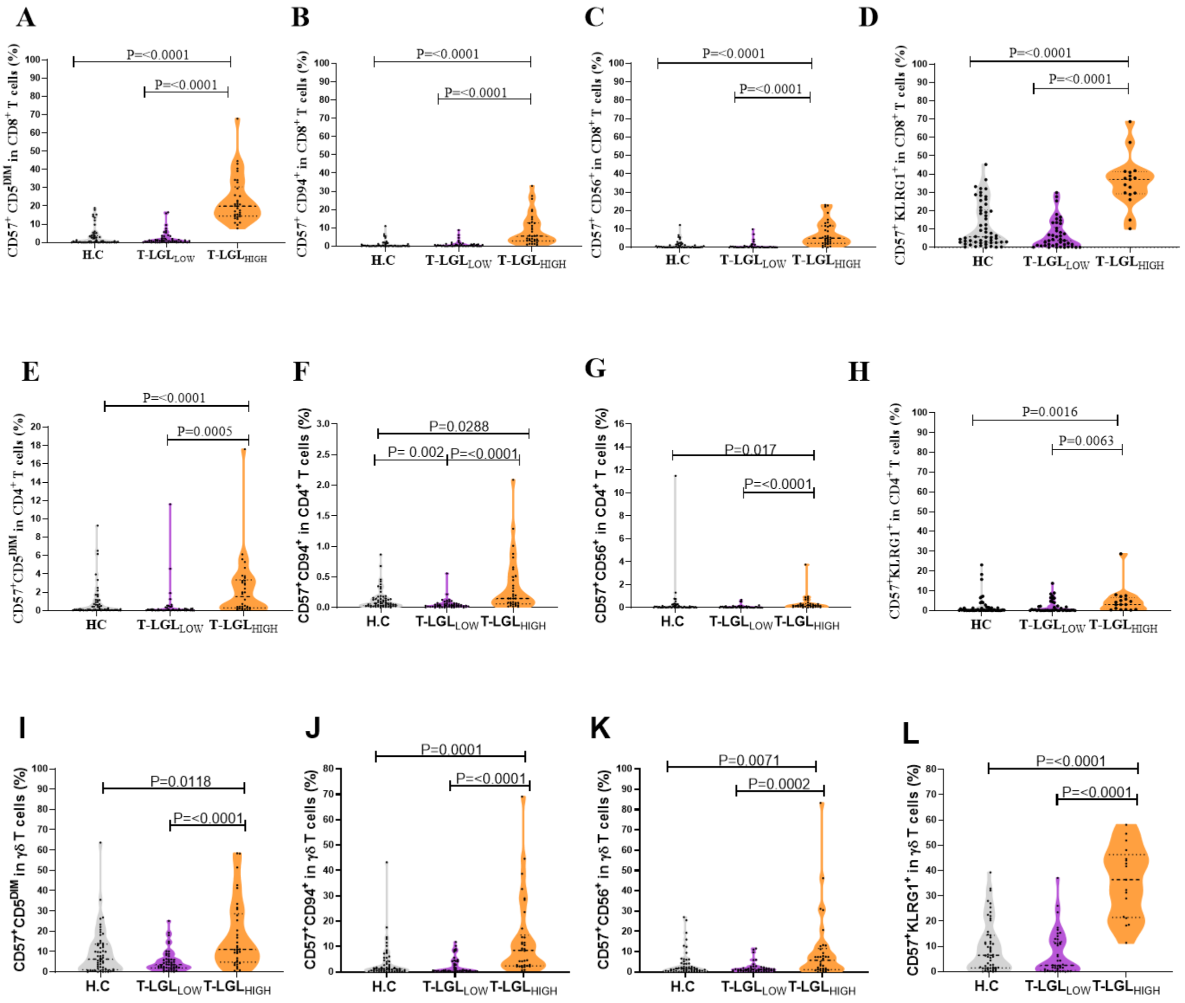

Supplementary Figure 6

Proportion of differentiation marker-expressing lymphocytes within the $CD8^+CD57^+$, $CD4^+CD57^+$ and $\gamma\delta^+CD57^+$ T cell populations. 56 HC, 51 IBM $T\text{-}LGL_{LOW}$ and 34 IBM $T\text{-}LGL_{HIGH}$ patients were analysed in this figure **A--D.** The proportion of $CD5^{DIM}$, CD94, CD56, gated on $CD8^+CD57^+$T cells between. Statistical analysis was performed using Kruskal-Wallis ANOVA with Dunn's post-hoc test for multiple comparisons. **E-H.** The proportion of $CD5^{DIM}$, CD94, CD56, gated on $CD4^+CD57^+$T cells. Statistical analysis was performed using Kruskal-Wallis ANOVA with Dunn's post-hoc test for multiple comparisons. **I-L.** The proportion of $CD5^{DIM}$, CD94, CD56, gated on $\gamma\delta^+CD57^+$T cells. Statistical analysis was performed using Kruskal-Wallis ANOVA with Dunn's post-hoc test for multiple comparisons.

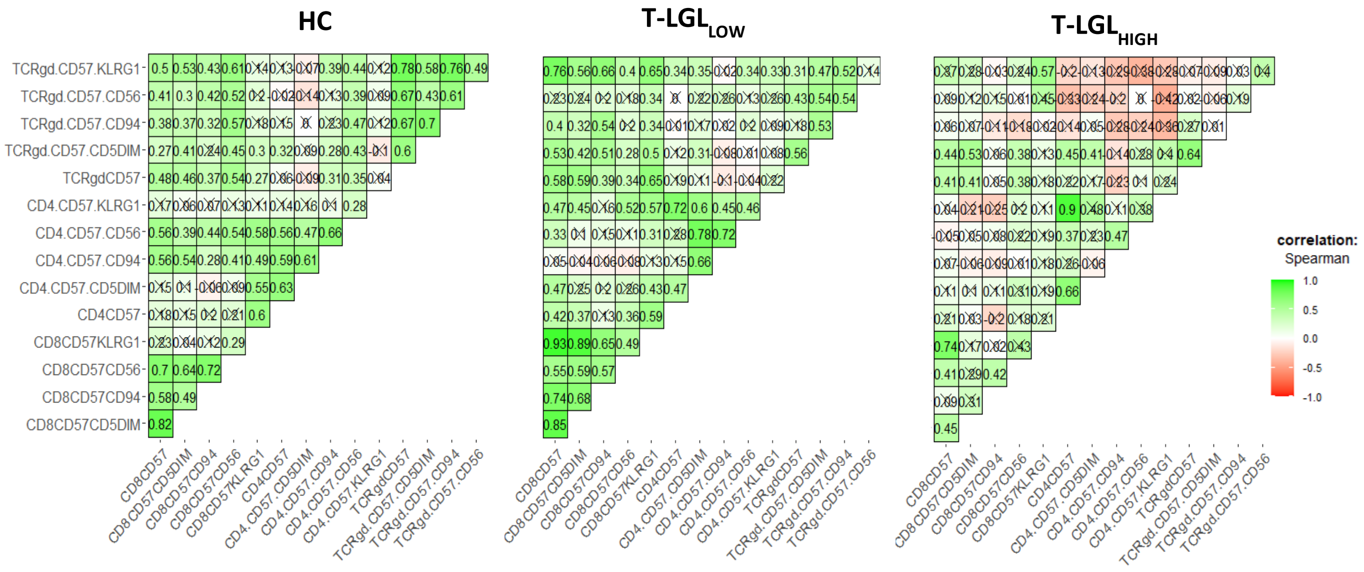

Supplementary Figure 7

Spearman's correlation matrix of phenotype markers in CD4+ CD8+ and TCRγδ CD57+ populations in HC (n=54), T-LGL$_{LOW}$ (n=50) and T-LGL$_{HIGH}$ (n=34). The values indicate the Spearman's correlation coefficient. X indicates correlation is not significant (P>0.05).

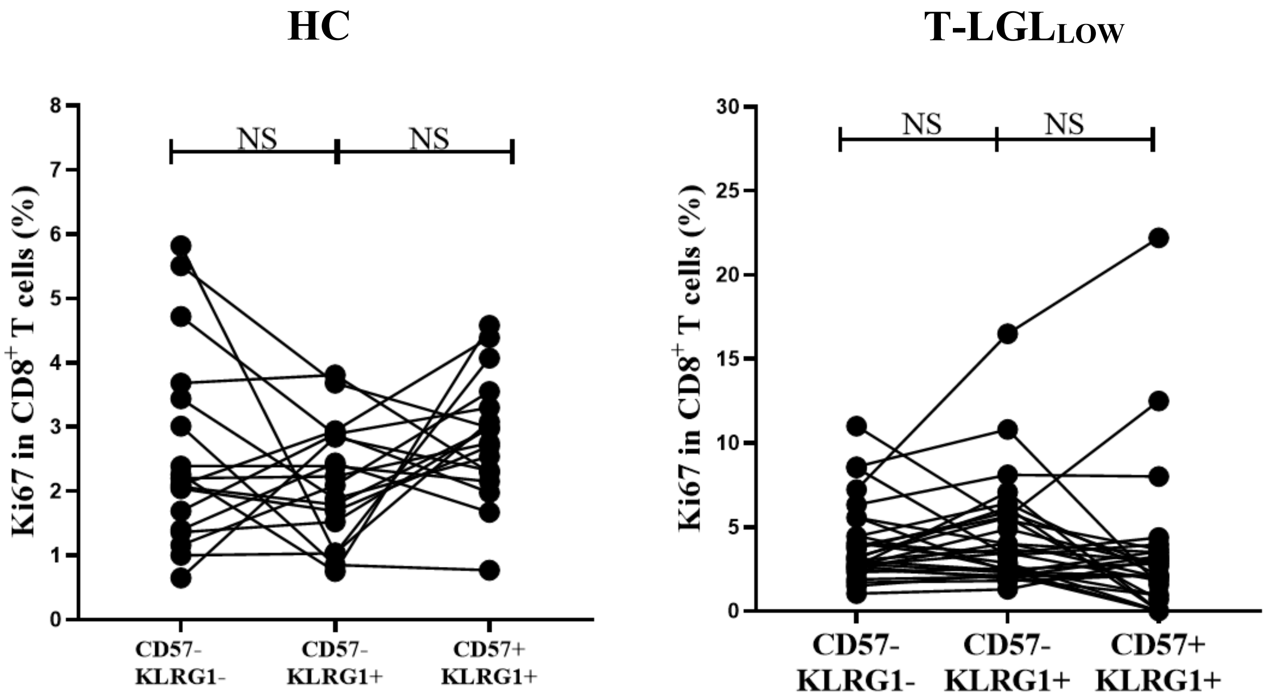

Supplementary Figure 8

Graphical representations of Ki67 in $CD8^+$ $CD57^-$ $KLRG1^-$ $CD8^+CD57^-KLRG1^+$ and $CD8^+$ $CD57^+KLRG1^+$ in HC (n=18) and IBM T-LGL_{LOW} (n=29), Statistical analysis was performed using Friedman test with Dunn's post-hoc test for multiple comparisons.

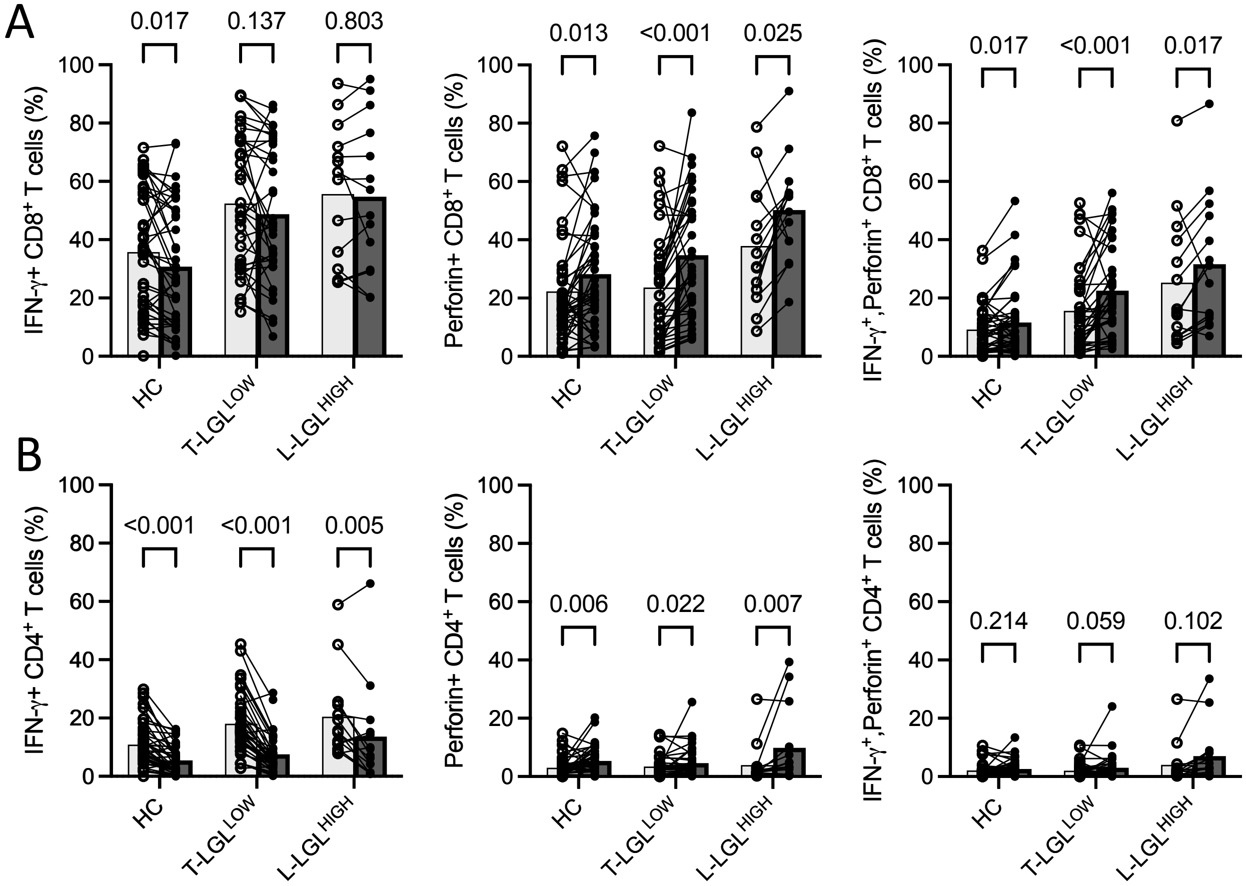

Supplementary Figure 9: IFN-γ and perforin content within CD8+ T cell subsets in IBM and HC donor groups. The frequency of CD8+ T cells producing IFN-γ (Left panel), perforin (middle panel) or co-producing both immune mediators was measured within the CD45RA-negative (light bars, open symbols) and -positive (dark bars, dark symbols) subsets in HC (n=41) and IBM LGL^{LOW} (n=36) and LGL^{HIGH} (n=14) donor groups. Linked symbols show the frequencies measured in each subset within a same sample. Bars show the mean values of individual measures. The significance of the differences was analysed using the Wilcoxon matched-paired signed rank test adjusted using the Benjamini, Krieger and Yekutieli method to account for false discovery rate due to multiple comparison analysis (calculated on GraphPad Prism 9.5.1); P-values are indicated above the respective bar pairs.

Supplementary Table 3: All HLA Allele Frequencies (>1%) Between IBM T-LGL$_{NEG}$ and IBM T-LGL$_{POS}$ Patients

	HLA ALLELE	% IN TOTAL N=144	% IN T-LGL$_{POS}$	% IN T-LGL$_{NEG}$	P.VALUE	P. ADJUSTED	ODD RATIO	95% CI LOW	95% CI HIGH
**1**	C*14:02:01	6.94%	12.07%	3.49%	0.05	1	4.24	1.07	21.24
**2**	DPB1*04:02:01	11.81%	17.24%	8.14%	0.08	1	2.71	0.90	8.57
**3**	DPB1*03:01:01	6.94%	1.72%	10.47%	0.10	1	0.17	0.01	0.90
**4**	C*12:03:01	3.47%	6.90%	1.16%	0.10	1	6.72	0.93	135.45
**5**	B*40:01:01	3.47%	6.90%	1.16%	0.10	1	6.72	0.93	135.45
**6**	C*03:04:01	3.47%	6.90%	1.16%	0.10	1	6.72	0.93	135.45
**7**	DQA1*05:01:01	42.36%	48.28%	38.37%	0.12	1	2.17	0.85	6.24
**8**	A*02:01:01	31.25%	37.93%	26.74%	0.16	1	1.69	0.82	3.59
**9**	C*04:01:01	8.33%	12.07%	5.81%	0.17	1	2.42	0.69	9.06
**10**	DPB1*13:01:01	2.78%	5.17%	1.16%	0.18	1	4.85	0.59	100.83
**11**	DRB1*11:01:01	4.17%	6.90%	2.33%	0.19	1	3.28	0.60	24.92
**12**	C*07:02:01	11.11%	6.90%	13.95%	0.23	1	0.50	0.14	1.42
**13**	A*24:02:01	4.86%	1.72%	6.98%	0.24	1	0.29	0.02	1.52
**14**	DRB1*01:03:01	4.17%	1.72%	5.81%	0.25	1	0.27	0.01	1.81
**15**	DRB3*02:02:01	22.92%	27.59%	19.77%	0.32	1	1.45	0.70	3.06
**16**	DQA1*01:02:01	7.64%	5.17%	9.30%	0.35	1	0.50	0.10	1.94
**17**	A*68:01:02	3.47%	1.72%	4.65%	0.36	1	0.35	0.02	2.51
**18**	B*14:02:01	2.08%	3.45%	1.16%	0.36	1	3.11	0.28	68.88
**19**	C*08:02:01	2.08%	3.45%	1.16%	0.36	1	3.11	0.28	68.88
**20**	B*57:01:01	2.08%	3.45%	1.16%	0.36	1	3.11	0.28	68.88
**21**	DPB1*02:02:01	2.08%	3.45%	1.16%	0.36	1	3.11	0.28	68.88
**22**	B*07:02:01	11.81%	8.62%	13.95%	0.37	1	0.62	0.19	1.67
**23**	C*07:01:01	27.78%	24.14%	30.23%	0.38	1	0.69	0.29	1.57
**24**	DQB1*03:01:01	9.72%	12.07%	8.14%	0.41	1	1.64	0.50	5.40
**25**	DPB1*01:01:01	9.72%	12.07%	8.14%	0.45	1	1.52	0.50	4.68
**26**	B*18:01:01	4.86%	3.45%	5.81%	0.51	1	0.56	0.08	2.83

27	DRB1*15:01:01	4.86%	3.45%	5.81%	0.51	1	0.56	0.08	2.83
28	DRB4*01:01:01	9.03%	6.90%	10.47%	0.51	1	0.69	0.19	1.99
29	DRB1*01:01:01	16.67%	18.97%	15.12%	0.52	1	1.36	0.53	3.50
30	DQA1*02:01:01	2.78%	1.72%	3.49%	0.53	1	0.48	0.02	3.94
31	DRB1*07:01:01	2.78%	1.72%	3.49%	0.53	1	0.48	0.02	3.94
32	B*44:03:01	2.78%	1.72%	3.49%	0.53	1	0.48	0.02	3.94
33	DPB1*05:01:01	2.78%	1.72%	3.49%	0.53	1	0.48	0.02	3.94
34	DRB1*15:02:01	2.78%	1.72%	3.49%	0.53	1	0.48	0.02	3.94
35	DRB5*01:01:02	2.78%	1.72%	3.49%	0.53	1	0.48	0.02	3.94
36	C*01:02:01	2.78%	1.72%	3.49%	0.53	1	0.48	0.02	3.94
37	A*11:01:01	5.56%	6.90%	4.65%	0.55	1	1.56	0.34	7.15
38	A*03:01:01	16.67%	18.97%	15.12%	0.56	1	1.28	0.54	3.03
39	B*08:01:01	24.31%	22.41%	25.58%	0.60	1	0.78	0.30	1.99
40	B*27:05:02	4.17%	5.17%	3.49%	0.61	1	1.54	0.27	8.87
41	B*51:01:01	9.03%	10.34%	8.14%	0.63	1	1.34	0.39	4.54
42	A*01:01:01	24.31%	22.41%	25.58%	0.65	1	0.83	0.35	1.87
43	DQA1*03:01:01	6.25%	5.17%	6.98%	0.65	1	0.71	0.14	2.96
44	DQB1*03:03:02	2.78%	3.45%	2.33%	0.69	1	1.52	0.17	13.28
45	B*35:01:01	7.64%	8.62%	6.98%	0.70	1	1.28	0.34	4.73
46	C*06:02:01	4.17%	3.45%	4.65%	0.72	1	0.72	0.10	3.98
47	DRB1*04:01:01	4.17%	3.45%	4.65%	0.72	1	0.72	0.10	3.98
48	C*05:01:01	9.72%	8.62%	10.47%	0.72	1	0.82	0.24	2.44
49	DRB3*01:01:02	32.64%	31.03%	33.72%	0.72	1	0.87	0.40	1.85
50	B*44:02:01	7.64%	6.90%	8.14%	0.79	1	0.85	0.22	2.79
51	DPB1*02:01:02	14.58%	15.52%	13.95%	0.80	1	1.13	0.44	2.82
52	DQB1*04:02:01	2.08%	1.72%	2.33%	0.80	1	0.73	0.03	8.00
53	DQB1*03:02:01	2.08%	1.72%	2.33%	0.80	1	0.73	0.03	8.00
54	DPB1*11:01:01	2.08%	1.72%	2.33%	0.80	1	0.73	0.03	8.00
55	DQA1*01:01:01	21.53%	20.69%	22.09%	0.82	1	0.90	0.36	2.22
56	DRB1*13:01:01	13.19%	13.79%	12.79%	0.85	1	1.11	0.37	3.20
57	B*15:01:01	5.56%	5.17%	5.81%	0.87	1	0.88	0.17	3.89
58	DRB5*01:01:01	5.56%	5.17%	5.81%	0.87	1	0.88	0.17	3.89
59	DQA1*01:03:01	16.67%	17.24%	16.28%	0.87	1	1.08	0.41	2.76

**60**	DQB1*06:02:01	4.86%	5.17%	4.65%	0.88	1	1.13	0.21	5.51
**61**	DPB1*04:01:01	36.81%	36.21%	37.21%	0.89	1	0.95	0.44	2.03
**62**	DQB1*05:01:01	20.83%	20.69%	20.93%	0.97	1	0.98	0.39	2.43
**63**	DQB1*06:03:01	13.89%	13.79%	13.95%	0.98	1	0.98	0.33	2.80
**64**	DQB1*02:01:01	36.11%	36.21%	36.05%	0.98	1	1.01	0.40	2.61
**65**	C*03:03:01	6.94%	6.90%	6.98%	0.98	1	0.99	0.23	3.81
**66**	C*15:02:01	2.78%	0.00%	4.65%	0.99	1	0.00	NA	$3.96x10^{52}$
**67**	DQB1*06:01:01	3.47%	3.45%	3.49%	0.99	1	0.99	0.12	6.34
**68**	C*02:02:02	3.47%	3.45%	3.49%	0.99	1	0.99	0.12	6.34
**69**	DPB1*10:01:01	3.47%	3.45%	3.49%	0.99	1	0.99	0.12	6.34
**70**	DPB1*19:01:01	2.08%	0.00%	3.49%	0.99	1	0.00	NA	$2.33\ x10^{71}$
**71**	A*26:01:01	2.08%	0.00%	3.49%	0.99	1	0.00	NA	$2.33\ x10^{71}$
**72**	B*52:01:01	2.08%	0.00%	3.49%	0.99	1	0.00	NA	$2.33\ x10^{71}$
**73**	C*12:02:01	2.08%	0.00%	3.49%	0.99	1	0.00	NA	$2.33\ x10^{71}$
**74**	A*29:02:01	2.08%	0.00%	3.49%	0.99	1	0.00	NA	$2.33\ x10^{71}$
**75**	C*16:01:01	2.08%	0.00%	3.49%	0.99	1	0.00	NA	$2.33x10^{71}$
